# Alzheimer’s disease pathological lesions activate the spleen tyrosine kinase

**DOI:** 10.1186/s40478-017-0472-2

**Published:** 2017-09-06

**Authors:** Jonas Elias Schweig, Hailan Yao, David Beaulieu-Abdelahad, Ghania Ait-Ghezala, Benoit Mouzon, Fiona Crawford, Michael Mullan, Daniel Paris

**Affiliations:** 1 The Roskamp Institute, 2040 Whitfield Avenue, Sarasota, FL 34243 USA; 2 The Open University, Milton Keynes, MK7 6AA UK; 3 James A. Haley Veterans’ Hospital, Tampa, FL 33612 USA

**Keywords:** Alzheimer’s disease, Spleen tyrosine Kinase, Dystrophic neurite, Aβ, sAPPβ, BACE1, Tau hyperphosphorylation, Tau oligomers, Tg PS1/APPsw, Tg APPsw, Tg Tau P301S

## Abstract

The pathology of Alzheimer’s disease (AD) is characterized by dystrophic neurites (DNs) surrounding extracellular Aβ-plaques, microgliosis, astrogliosis, intraneuronal tau hyperphosphorylation and aggregation. We have previously shown that inhibition of the spleen tyrosine kinase (Syk) lowers Aβ production and tau hyperphosphorylation in vitro and in vivo*.* Here, we demonstrate that Aβ-overexpressing Tg PS1/APPsw, Tg APPsw mice, and tau overexpressing Tg Tau P301S mice exhibit a pathological activation of Syk compared to wild-type littermates. Syk activation is occurring in a subset of microglia and is age-dependently increased in Aβ-plaque-associated dystrophic neurites of Tg PS1/APPsw and Tg APPsw mice. In Tg Tau P301S mice, a pure model of tauopathy, activated Syk occurs in neurons that show an accumulation of misfolded and hyperphosphorylated tau in the cortex and hippocampus. Interestingly, the tau pathology is exacerbated in neurons that display high levels of Syk activation supporting a role of Syk in the formation of tau pathological species in vivo. Importantly, human AD brain sections show both pathological Syk activation in DNs around Aβ deposits and in neurons immunopositive for pathological tau species recapitulating the data obtained in transgenic mouse models of AD. Additionally, we show that Syk overexpression leads to increased tau accumulation and promotes tau hyperphosphorylation at multiple epitopes in human neuron-like SH-SY5Y cells, further supporting a role of Syk in the formation of tau pathogenic species. Collectively, our data show that Syk activation occurs following Aβ deposition and the formation of tau pathological species. Given that we have previously shown that Syk activation also promotes Aβ formation and tau hyperphosphorylation, our data suggest that AD pathological lesions may be self-propagating via a Syk dependent mechanism highlighting Syk as an attractive therapeutic target for the treatment of AD.

## Introduction

Alzheimer’s disease (AD) is a neurodegenerative disease that accounts for the majority of all cases of dementia. AD pathological hallmarks include extracellular aggregates of Aβ, intracellular tau hyperphosphorylation and aggregation, as well as neuroinflammation. Tau is a microtubule-associated protein (MAP) involved in many essential cellular processes including stabilization of the microtubule network, thereby providing a functional basis for intracellular transport [10]. Misfolding and pathological post-translational modifications including tau hyperphosphorylation contribute to its oligomerization and accumulation that ultimately leads to neuronal death [10]. In addition, tau mutations that cause familial forms of dementia associated with the formation of tau aggregates have been identified suggesting that pathological tau species may play a key role in AD.

Tau and Aβ have been proposed to synergistically contribute to the pathobiology of AD [25]. Through cleavage of the amyloid precursor protein (APP) by α, β and γ-secretases different variants of Aβ and soluble APP forms (α, β) are generated [41]. A variety of post-translational modifications and the nature of the Aβ variants define their susceptibility to aggregation and neurotoxicity [41, 42]. Several mutations in the *APP* and presenilin (*PSEN1/2)* genes (members of the γ-secretase complex) have been identified and cause familial forms of AD (FAD) [36]. These mutations either render APP more susceptible to cleavage by the β-secretase (BACE-1) or the γ-secretase resulting in increased Aβ production or lead to the production of longer forms of Aβ that are more prone to aggregation and accumulation resulting in early onset AD (EOAD). In contrast, the etiology of sporadic or late onset AD (LOAD) accounts for more than 99% of all AD cases and remains unknown [24].

Many studies have suggested the importance of neuroinflammation caused by Aβ in AD and that a therapeutic strategy can only be successful if it counteracts the neurotoxicity caused by inflammation [24, 29]. Aβ fibrils have been shown to trigger an inflammatory response in primary microglial and monocytic cells via an activation of the tyrosine kinases Lyn (**L**ck/**Y**es **n**ovel tyrosine kinase) and Syk (spleen tyrosine kinase) [3, 23]. Importantly, Syk inhibition appears to prevent Aβ-mediated neurotoxicity in vitro [3]. A subsequent study also showed that Syk is the mediator of the Aβ-induced cytokine production including tumor necrosis factor alpha (TNFα) and interleukin 1 beta (IL-1β) by activated microglia [4] suggesting that Syk is a key kinase responsible for the proinflammatory activity of Aβ.

Many different sites of tau hyperphosphorylation have been identified in AD and various kinases have been the subject of investigations regarding their possible involvement in tau pathogenesis. Syk and Src family kinases have been shown to phosphorylate tau directly at Y18 [20, 25]. Tau tyrosine phosphorylation is considered an early pathological change in AD [5, 20]. Syk has also been shown to phosphorylate microtubules which could have an effect on microtubule polymerization or the interaction of signaling molecules with the microtubule network [6]. Moreover, pharmacological Syk inhibition has been found to stabilize microtubules through dephosphorylation of microtubules and microtubule associated proteins (MAPs) [44].

We have previously shown that Syk regulates the activation of the glycogen synthase kinase-3β (GSK3β), one of the main tau kinase that phosphorylates tau at multiple sites present in neurofibrillary tangles [28]. In addition, we have shown that Syk also regulates Aβ production and proposed that Syk could be an important therapeutic target for the treatment of AD as pharmacological inhibition of Syk appears to reduce tau hyperphosphorylation and Aβ production both in vitro and in vivo [28].

Syk is a non-receptor protein-tyrosine kinase (PTK) that mediates inflammatory responses [8]. PTKs like Syk are part of receptor-mediated signal transduction cascades that require their intracellular association with integral membrane receptors including toll-like receptors (TLRs [11]) and Fc receptors (FcγR [14], FcεRI [21]). Recruitment and activation of Syk is also mediated by activation of triggering receptor expressed on myeloid cells 2 (TREM2) [18]. Interestingly, several variants of TREM2 are associated with an increased risk to develop AD and have been shown to alter AD pathology including Aβ deposition, tau hyperphosphorylation, neuroinflammation and synaptic loss in AD mouse models [17]. Syk becomes active through autophosphorylation and several Syk autophosphorylation sites have been identified in vitro: Y130, Y290, Y317, Y346, Y358, and Y525/526. The Y525/526 phosphorylation site is the main site involved in receptor-mediated Syk activation and signal propagation [30]. Although our previous work suggests that Syk could represent a therapeutic target for AD, the cellular localization and the activity pattern of Syk in the brains of transgenic mouse models of AD and AD pathological specimens remains to be determined. We therefore investigated in this study whether Syk activation occurs in the brains of different mouse models of AD and in human AD brain by monitoring the Y525/526 Syk autophosphorylation site and analyzing its association with AD pathological hallmarks.

We investigated two different AD mouse models that overexpress APP and one mouse model of pure tauopathy that overexpresses human tau with the P301S mutation. In our study, we employed transgenic APPsw (Tg 2576) mice overexpressing the Swedish mutation (KM670/671NL) of APP695 under the control of the hamster prion protein promoter [13]. These mice have elevated levels of Aβ and typically develop Aβ plaques at the age of 11 months [15]. We also analyzed transgenic PS1/APPsw mice which carry the APP KM670/671NL (Swedish) and the PSEN1 M146L mutations. In these mice, the human PSEN1 M146 L transgene is driven by the PDGF-β promoter. These double transgenic mice develop cortical and hippocampal amyloid deposits at 6 months of age; much earlier than the single transgenic APPsw (Tg2576). Additionally, the total Aβ burden is increased in these double transgenic mice compared to the single Tg 2576 transgenic mice [12]. Aβ deposits are associated with dystrophic neurites that occur at 12 months of age in Tg PS1/APPsw mice [9]. Furthermore, these mice display an increase in Aβ plaque-associated microglia and astrocytes at 6 months of age. However, increased microglial activity has been found to occur at 12 months [9]. In addition, we analyzed whether Syk activation occurs in the brain of transgenic Tau P301S PS19 mice that overexpress human tau with the P301S mutation. The P301S mutation in the tau gene on chromosome 17 has been associated with autosomal dominantly inherited frontotemporal dementia and parkinsonism (FTDP-17) [1, 22, 38]. The expression of the P301S mutated tau is fivefold higher in Tg Tau P301S mice than the endogenous mouse protein and is driven by the mouse prion protein promoter [43]. Interestingly, these mice progressively develop neurodegeneration and display intraneuronal tau hyperphosphorylation and aggregation that closely mimic neurofibrillary tangles.

In this study, we show by high-resolution confocal microscopy that Syk activation is increased in a subset of activated microglia and in dystrophic neurites around Aβ plaques of Tg APPsw and Tg PS1/APPsw mice. Interestingly, pSyk is also age-dependently increased in neurons of Tg Tau P301S mice. The degree of colocalization between Syk and tau is largely dependent on the tau epitope investigated and differs between various phospho-tau epitopes and tau oligomers/conformers. The level of Syk activation, as measured by fluorescence intensity, correlates with the amount of pathological tau species detected. In addition, we show that Syk overexpression in human neuronal like cells (SH-SY5Y) results in increased total tau and tau phosphorylation levels at multiple epitopes. Taken together, our results show that β-amyloid and tau pathological species both activate Syk in vivo and conversely, that Syk is involved in microglial activation, plays a role in the pathogenesis of dystrophic neurites (DNs) and contributes to the formation of pathological tau species therefore exacerbating AD pathological lesions. Interestingly, human AD brain sections exhibit the same pattern of Syk activation as the mouse models of β-amyloidosis and tauopathy combined. Human AD brain sections show an increase in pSyk (phosphorylated Syk at Y525/526) levels in DNs around β − amyloid plaques and in neurons immunopositive for hyperphosphorylated tau (Y18) and pathological tau conformers (MC1), whereas brain sections from non-demented controls do not show any pSyk increase. Altogether, these data suggest a crucial role of Syk in the pathobiology of AD and highlight Syk as a promising therapeutic target in AD.

## Materials and methods

### Animals

Tg PS1/APPsw, Tg APPsw, Tg Tau P301S and wild-type mice were generated and maintained in a C57BL/6 genetic background as previously described [28]. All mice were maintained under specific pathogen free conditions in ventilated racks in the Association for Assessment and Accreditation of Laboratory Animal Care International (AAALAC) accredited vivarium of the Roskamp Institute. All experiments involving mice were reviewed and approved by the Institutional Animal Care and Use Committee of the Roskamp Institute before implementation and were conducted in compliance with the National Institutes of Health Guidelines for the Care and Use of Laboratory Animals.

### Tissue processing

All mice were humanely euthanatized and their brains were collected and fixed in 4% paraformaldehyde (PFA) for 48 h. The method of euthanasia used follow the AVMA (American Veterinary Medical Association) guidelines for the euthanasia of animals. Briefly, mice were rendered unconscious through inhalation of 5% isoflurane in oxygen using a vaporizer and a gas chamber. While under anesthesia, after verifying the absence of reflexes, mice were euthanatized by exsanguination (blood was withdrawn from cardiac puncture).

Subsequently, the hemispheres were processed in a Sakura Tissue-Tek VIP (Leica Biosystems Inc., IL, USA) vacuum infiltration processor. Brains were then embedded in paraffin with the Sakura Tissue-Tek (Leica Biosystems Inc., IL, USA) and stored at 4 °C for 2 days for subsequent cutting with a Leica RM2235 microtome (Leica Biosystems Inc., IL, USA). All brains were cut at a thickness of 12 μm. Sagittal slices were mounted on glass slides and dried for 48 h at 37 °C for subsequent immunofluorescence staining and confocal imaging.

### Immunofluorescence

Paraffin sections were washed in two baths of histoclear (National Diagnostics, USA) and progressively rehydrated with ethanol gradients and phosphate buffered saline (PBS, Sigma Aldrich, MO, USA). Brain sections were subjected to antigen retrieval for 7 min in citric acid buffer (pH 6) at 100 °C. All sections were treated with 0.05% Sudan Black in 70% ethanol to quench autofluorescence. Sections were then blocked in PBS containing 10% donkey serum (Abcam, MA, USA) for 1 h. Sections were incubated in PBS containing 1% donkey serum and the respective panel of primary antibodies overnight at 4 °C. The following antibodies were used: CP13 (anti(α)-phospho-tau (pTau) S202, 1:200, Dr. Peter Davies’ Lab), MC1 (α-conformational tau, 1:200, Dr. Peter Davies’ Lab), TOC1 (1:200, Dr. Lester Binder’s Lab), PHF-1 (α-pTau S396/404, 1:200, Dr. Peter Davies’ Lab), 9G3 (α-pTau Y18, 1:200, MediMabs Inc., QC, Canada), DA9 (α-total-tau (tTau), 1:200, Dr. Peter Davies’ Lab), α-BACE1 (1:200 Cell Signaling, MA, USA), α-sAPPβ with Swedish mutation (1:100 Immuno-Biological Laboratories Co, Ltd., Japan), α-Iba1 (1:300, Abcam, MA, USA), α-GFAP (1:5000, Aves Labs, OR, USA), α-pSyk (Y525/526, 1:200, Cell Signaling, MA, USA). In addition to the α-pSyk (Y525/526, 1:200, Cell Signaling, MA, USA), we used the α-pSyk (Y525/526, 1:100, Abgent, CA, USA) and obtained similar results. After three washing steps in PBS for 5 min, sections were incubated in a solution containing PBS, 1% donkey serum and the respective panel of secondary antibodies for 1 h in the dark at room temperature in a humidified chamber. The following secondary antibodies were used: donkey α-rabbit, α-goat, α-mouse conjugated to Alexa 488, 568 and 647, respectively (1:500, Life technologies). After three washing steps in PBS for 5 min, sections were mounted in Fluoroshield with or without DAPI (Sigma Aldrich, MO, USA). All images were acquired using the confocal microscope LSM 800 (Carl Zeiss AG, Germany), the ZEN Blue 2.1 (Carl Zeiss AG, Germany) software and a 20× or 63× objective. The acquisition settings were kept the same for all genotypes within the same experiment.

**Fig. 1:**
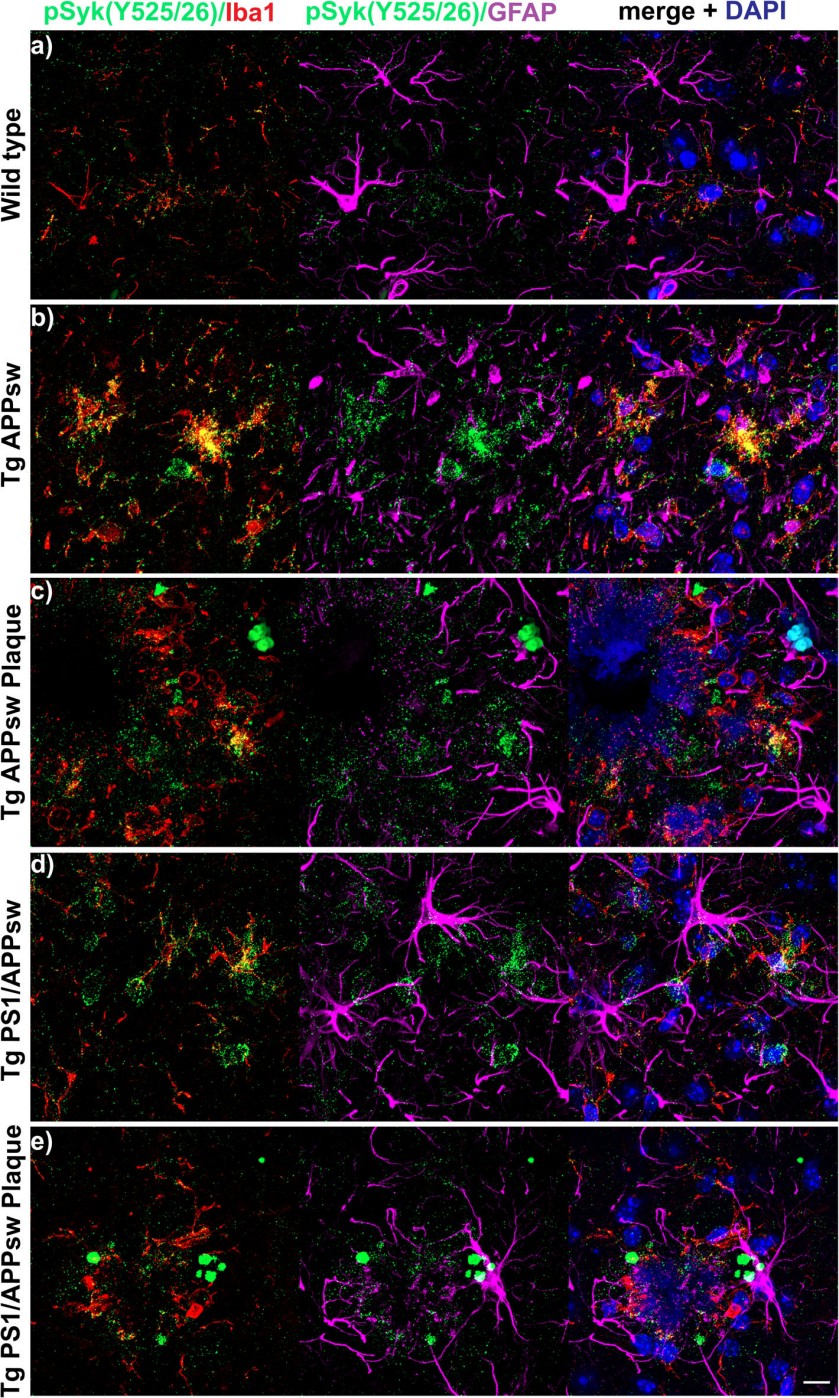
pSyk is increased in activated microglia and non-glial cells associated with Aβ-plaques in Tg APPsw and Tg PS1/APPsw mice. **a** Spatial distribution and cellular localization of activated/phosphorylated Syk were investigated in the cortex of 116 ± 13.5-week-old (avg. ± SEM) wild-type mice (*n* = 6) by triple-immunostaining of pSyk (Y525/526, green), microglia (Iba1, red) and astrocytes (GFAP, purple). Nuclei were stained with DAPI (blue). **b** Syk activation in wild-type animals was compared to age-matched Tg APPsw (*n* = 6) (**b**-**c**) and Tg PS1/APPsw littermates (*n* = 6) (**d**-**e**). Plaque-associated cortical areas (**c**, **e**) were compared to non-plaque-associated areas (**b**, **d**). Qualitative image analysis of orthogonal projections and 3D-image analysis (not-shown) revealed an increased pSyk burden in transgenic (**b**-**e**) compared to wild-type mice (**a**) and a colocalization of pSyk and Iba1 but not GFAP in Aβ-overexpressing animals (**b**-**d**). Large, non-glial spherical accumulations of pSyk were observed in plaque-associated areas (**e**). The scale bar represents 10 μm

For qualitative analysis of the pSyk burden in Tg PS1/APPsw and Tg APPsw mice compared to age-matched WT littermates (*n* = 6 for each genotype, equal amount of male and female), 116 ± 13.5 (avg. ± SEM) weeks of age were stained and analyzed as described above (Fig. 1).

For qualitative analysis of the pSyk burden in Tg Tau P301S mice compared to WT littermates, hippocampi and cortices of 16 male and female mice ranging from 8 to 56 weeks of age were stained and analyzed as described above.

For the quantitative analysis of the pSyk burden (Fig. 3), 140 randomly-selected microscopic fields of four non-consecutive brain slices (containing the hippocampus) from six animals per genotype (equal number of male and female) were acquired. The area covered with the pSyk immunopositive staining was quantified with Fiji [34] in microscopic fields containing Aβ plaques as well as in microscopic fields not containing Aβ deposits. The PS1/APPsw, APPsw and WT mice of the younger cohort were on average 45 ± 0.3 (avg. ± SEM) weeks old. The average age of the mice of the older cohort was 116 ± 13.5 weeks (±SEM). The pSyk burden of the transgenic mice was normalized to the level of pSyk burden quantified in wild-type littermates of the respective age-group. As a negative control, primary antibodies were omitted to determine background and autofluorescence (not shown).

For the quantitative analysis of the colocalization of pSyk and different tau epitopes (Fig. 8) between 400 and 570 cortical fields (50,000 μm^2^ per field) from four male Tg Tau P301S animals (average age 47 ± 3.1 (SEM) weeks) were analyzed for each tau epitope. To quantify the percentage of the immunopositive neurons a total of 2546 microscopic fields and 21,800 neurons were counted using the Zen Blue 2.1 software (Carl Zeiss AG, Germany).

The fluorescence intensities (Figs. 9, 10, 11, 12 and 13) of 30 to 40 neurons immunopositive for pSyk, pTau or both (colocalized) were determined for each tau epitope (total of 90 neurons per epitope) using Zen Blue 2.1 (Carl Zeiss AG, Germany). The male Tg Tau P301S mice (*n* = 4) used for quantification were on average 47 ± 3.1 weeks old (avg. ± SEM).

In addition, the different immunostainings mentioned above were performed on paraffin-embedded tissue sections (10 μm, dorsolateral frontal cortex) from a 67-year-old, male patient with AD (Braak VI) and a 102-year-old, male non-demented control that were provided by Dr. Ann McKee (Boston University, MA, USA). Institutional review board approval for brain donation was obtained through the Boston University Alzheimer’s Disease Center (BUADC, Boston, MA, USA).

### Cell culture

SH-SY5Y cells were purchased from American Type Culture Collection (VA, USA). SH-SY5Y cells were grown in DMEM/F12 medium (Thermo Fisher Scientific, MA, USA) supplemented with 10% fetal bovine serum (Thermo Fisher Scientific, MA, USA), GlutaMAX and 1% penicillin/streptomycin/fungizone.

### Generation of Syk overexpressing SH-SY5Y cells

A human cDNA ORF Clone of the human SYK gene (NM_003177, transcript variant 1) was purchased from OriGene Technologies (MD, USA). The cDNA fragment encoding human SYK was amplified by PCR using PfuUltra II Fusion HS DNA polymerase (Agilent Genomics, CA, USA) and subcloned into the p3xFLAG-Myc-CMV™-26 Expression Vector (Sigma-Aldrich, MO, USA) to generate the pCMV-SYK-Flag plasmid. The entire reading frame of the plasmid was confirmed by DNA sequencing. SH-SY5Y cells were maintained in advanced DMEM/F-12 medium supplemented with 10% fetal bovine serum, 1% GlutaMAX, 1% penicillin/streptomycin (Thermo Fisher Scientific, MA, USA) and incubated in a humidified 5% CO_2_ atmosphere at 37 °C. For stable transfection, SH-SY5Y cells were grown in 6-wells cell culture plates until reaching 70-80% confluence and transfected with 3 μg of empty pCMV vector (control cells) or pCMV-SYK-Flag plasmids per well using lipofectamine 2000 (Thermo Fisher Scientific, MA, USA). After 48 h, the medium surrounding transfected cells was replaced with fresh medium containing 0.2 mg/ml of G418 for selection. After 14 days of selection, G418 resistant cells were trypsinized and expanded. The expression efficiency of SYK was analyzed by Western blot using antibodies against SYK (4D10 Syk antibody, Santa Cruz Biotechnology, TX, USA) and the Flag tag (Sigma-Aldrich, MO, USA).

### Immunoblotting

SH-SY5Y cells were cultured in 24-well-plates for 24 h and subsequently lysed with mammalian protein extraction reagent (MPER, Thermo Fisher Scientific, MA, USA) containing Halt protease & phosphatase single use inhibitor/EDTA (Thermo Fisher Scientific, MA, USA) and 1 mM PMSF. Proteins of cell lysates were separated by 10% tris-glycine-SDS-PAGE using 1 mm Criterion TGX gels (Bio-Rad Laboratories, CA, USA) and electro-transferred onto 0.2 μm PVDF membranes (Bio-Rad Laboratories, CA, USA). Membranes were blocked in TBS containing 5% non-fat dried milk for 1 h and were hybridized with the primary antibody (αSyk (4D10, 1:1000, Santa Cruz, TX, USA), αpTau S396/404 (PHF-1, 1:1000, Dr. Peter Davies’ Lab), αtTau (DA9, 1:1000, Dr. Peter Davies’ Lab), αpTau Y18 (9G3, 1:1000, MediMabs Inc., QC, Canada,) overnight at 4 °C. Subsequently, the membranes were incubated for 1 h in HRP-conjugated αmouse secondary antibody (1:1000, Cell Signaling, MA, USA). Western blots were visualized using chemiluminescence (Super Signal West Femto Maxium Sensitity Substrate, Thermo Fisher Scientific, MA, USA). Signals were quantified using ChemiDoc XRS (Bio-Rad Laboratories, CA, USA) and densitometric analyses were performed using Quantity One (Bio-Rad Laboratories, CA, USA) image analysis software.

### Statistical analyses

The data were analyzed and plotted with GraphPad Prism (GraphPad Software, Inc., CA, USA). The Shapiro-Wilk test for normality was used to test for Gaussian distribution. Statistical significance was determined by either Kruskal-Wallis followed by Dunn’s post-hoc test or the non-parametric Mann–Whitney test. All data are presented as mean ± the standard error of the mean (SEM) and *p* < 0.05 was considered significant.

## Results

### Syk activation in activated microglia and non-glial cells associated with Aβ-plaques in Tg APPsw and Tg PS1/APPsw mice

**Fig. 2:**
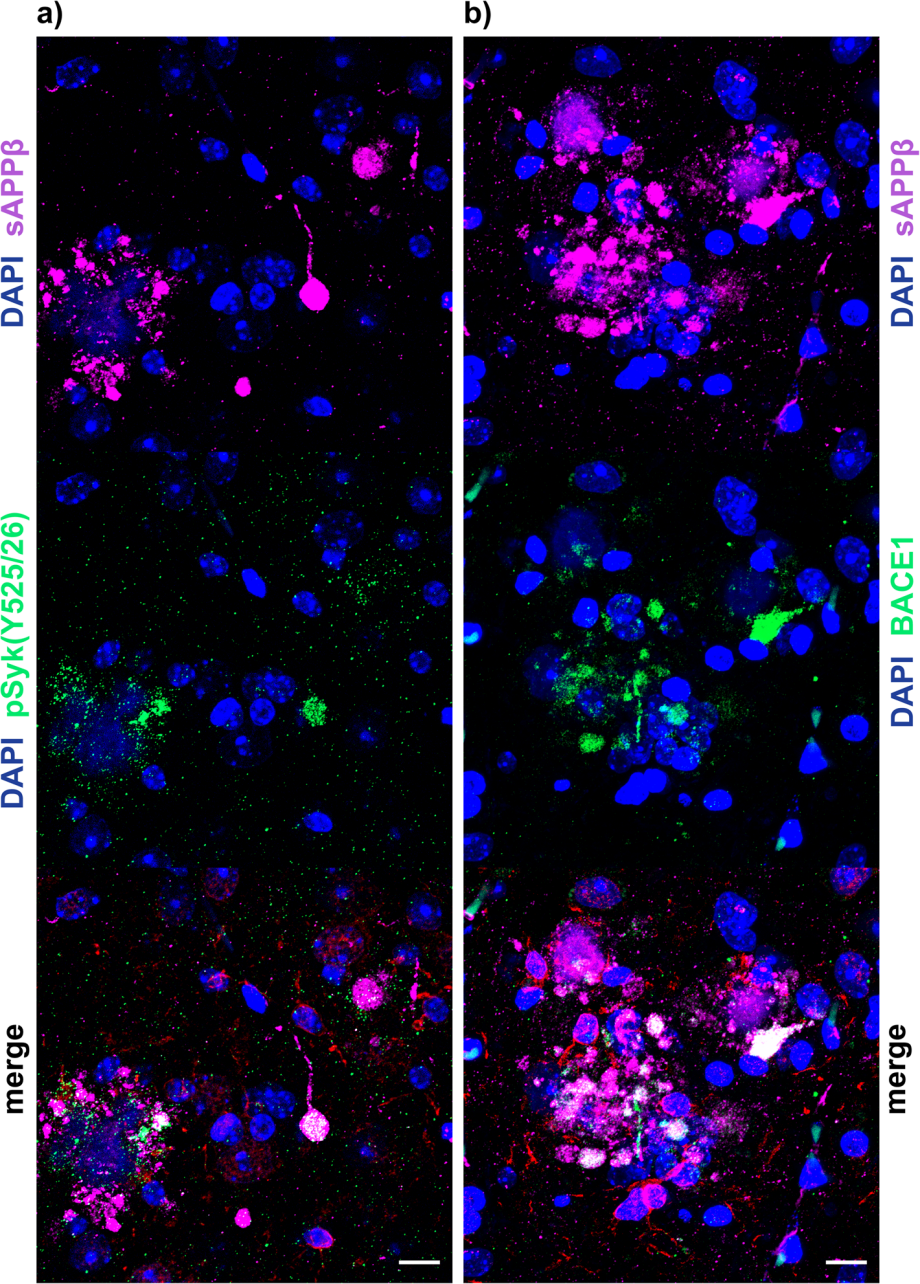
pSyk is increased in dystrophic neurites of Aβ-overexpressing mice. Representative confocal image of depicting the cortex of 116.5 ± 13.5-week-old Tg PS1/APPsw mice stained for sAPPβ (*purple*), pSyk (*green*, **a**), BACE1 (*green*, **b**), Iba-1 (*red*). Nuclei were stained with DAPI (*blue*). **a** pSyk was accumulated in dystrophic neurites positive for sAPPβ (**a**) and BACE1 (**b**). The scale bars represent 10 μm

To investigate whether pathological Syk activation occurs in the brain of AD mouse models, we analyzed the brains of 116-week-old wild-type, Tg APPsw and Tg PS1/APPsw mice using high-resolution confocal microscopy and immunofluorescence. All transgenic mice (Fig. 1b-e) exhibit an increased Iba-1 and GFAP reactivity compared to wild-type littermates (Fig. 1a). Moreover,wild-type some of the activated amoeboid microglia that are observed in transgenic mice are also strongly positive for pSyk (Fig. 1b-d). By contrast, we did not detect any pSyk immunoreactivity in astrocytes (Fig. 1). In addition, we observed that pSyk immunoreactivity is upregulated near Aβ plaques but neither colocalizes with microglia nor astrocytes suggesting that it could be of neuronal origin. (Fig. 1e). We further investigated the cellular origin of these pSyk accumulations by immunofluorescence staining and confocal microscopy (Fig. 2).

### pSyk is increased in dystrophic neurites of Aβ-overexpressing mice

To further characterize the cellular origin of pSyk accumulations near Aβ plaques, we tested different markers of dystrophic neurites (BACE-1 and sAPPβ) [31] and found a strong colocalization between pSyk and sAPPβ (Fig. 2a) associated with Aβ deposits. The sAPPβ staining clearly reveals dystrophic swellings of neurites (Fig. 2a) which are a known hallmark of AD. Most of the dystrophic neurites are positive for pSyk (Fig. 2a). Additionally, we found a strong colocalization between sAPPβ and BACE-1 (Fig. 2b) which are often used as markers of dystrophic neurites. Both sAPPβ and BACE-1 exhibit circular accumulations near Aβ plaques (Fig. 2b), highly reminiscent of the pattern observed for activated Syk.

In conclusion, activated Syk is not only found in microglia but also in neurons near Aβ deposits, particularly in dystrophic neurites of Tg APPsw and Tg PS1/APPsw mice supporting a possible role of Syk activation in the formation of dystrophic neurites. Dystrophic neurites are characterized by an accumulation of BACE-1 and sAPPβ [31] and our previous work [28] has shown that Syk regulates BACE-1 expression and sAPPβ levels suggesting that Syk upregulation in dystrophic neurites could contribute to the accumulation of BACE-1 and sAPPβ.

### Cortical pSyk burden is age-dependently increased in Aβ-overexpressing mice, particularly in microscopic fields containing Aβ-plaques, compared to wild-type littermates

**Fig. 3:**
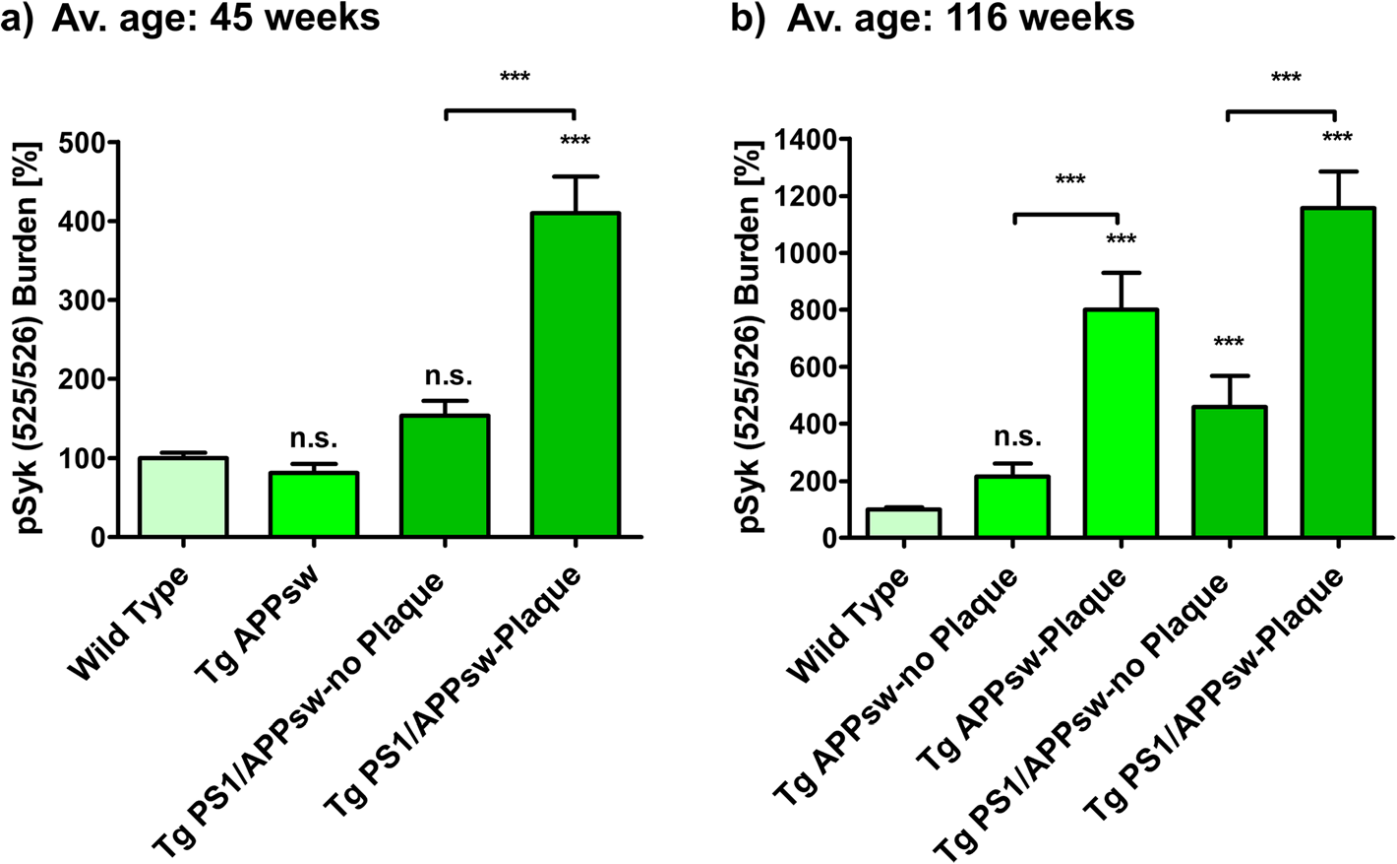
Cortical pSyk burden is age-dependently increased in Aβ-overexpressing mice, particularly in microscopic fields containing Aβ deposits, compared to wild-type littermates. Cortical pSyk burden (area covered) of immunofluorescence images (Fig. 1) was quantified in 45 ± 0.3-week-old (avg. ± SEM) (**a**) and 116 ± 13.5-week-old (avg. ± SEM) (**b**) Tg APPsw (*n* = 6) and Tg PS1/APPsw mice (*n* = 6), compared and normalized to wild-type littermates (*n* = 6). Microscopic fields containing Aβ deposits were distinguished from microscopic fields not containing Aβ deposits as described in the materials and methods section. Kruskal-Wallis and post-hoc Dunn’s multiple comparison test revealed a significant increase (*p* < 0.001) in pSyk in fields containing Aβ deposits in younger Tg PS1/APPsw animals compared to age-matched wild-type littermates (**a**). pSyk burden in older Tg APPsw and Tg PS1/APPsw mice was statistically significantly increased in cortical microscopic fields containing Aβ deposits compared to age-matched wild-type littermates (*p* < 0.001). Older Tg PS1/APPsw mice also exhibited a statistically significant pSyk burden increase in microscopic fields not containing Aβ deposits (*p* < 0.001), whereas the pSyk burden in Tg APPsw in microscopic fields not containing Aβ deposits was not statistically different from wild-type littermates (*P* > 0.05). Six animals per genotype were analyzed. Error bars represent SEM

We also quantified the pSyk burden observed in the cortex of Tg APPsw, Tg PS1/APPsw and wild-type (WT) littermates (Figs. 1 and 2). Two different age-groups were investigated: younger animals, 45 weeks of age and older animals 116 weeks of age in average. In 45-week-old Tg APPsw mice, we did not observe significant β-amyloidosis (only three Aβ plaques were found in the cohort of mice analyzed) (data not shown) showing that, at that age, the Aβ pathology is almost inexistent in these mice. We differentiated between microscopic fields containing Aβ deposits and microscopic fields not containing Aβ deposits for the quantification of the pSyk burden in Tg PS1/APPsw and older Tg APPsw mice. 45-week-old Tg APPsw and Tg PS1/APPsw mice do not show any significant difference in pSyk burden in fields without Aβ deposits compared to WT mice. The pSyk burden of 45-week-old Tg APPsw mice is identical to that of the WT mice (100 ± 6.76% compared to 80.85 ± 11.77%; Fig. 3a). The pSyk burden in fields not containing Aβ plaques in Tg PS1/APPsw mice is not statistically significantly elevated (153.48 ± 18.47%), compared to the WT littermates. As expected, 45-week-old Tg PS1/APPsw mice exhibited a significantly higher pSyk burden in fields containing Aβ plaques (410.19 ± 46.46%) compared to WT and Tg APPsw mice.

The analysis of the pSyk burden in the cortex of older animals (average age: 116 weeks) revealed large differences between genotypes. The pSyk burden of Tg APPsw (216.32 ± 45.23%) mice in microscopic fields without plaques is not significantly increased compared to WT mice (100 ± 7.78%) (Fig. 3b). In contrast, microscopic fields of older Tg APPsw mice containing Aβ deposits exhibit a strong increase in pSyk burden (799.95 ± 130.19%) compared to age-matched WT mice. Tg PS1/APPsw mice also exhibit a statistically significant increase in pSyk burden in microscopic fields that do not contain Aβ deposits (458.1 ± 109.68) compared to age-matched WT controls. In addition, a much greater pSyk burden is found in Tg PS1/APPsw in microscopic fields containing Aβ deposits. In these fields, the pSyk burden is increased by 1157.31 ± 129.68% compared to WT littermates (Fig. 3b).

In conclusion, our data show that the pSyk burden is highly associated with Aβ plaques and increases with age in Tg PS1/APPsw and Tg APPsw mice whereas no activation of Syk is observed in the brain of WT littermates. The upregulation of Syk activation observed in the brains of Tg APPsw and Tg PS1/APPsw is mainly attributable to pSyk accumulations in dystrophic neurites that are associated with Aβ plaques and increase with age and Aβ burden.

### Syk activity is increased in hippocampal and cortical neurons of Tg Tau P301S mice

**Fig. 4:**
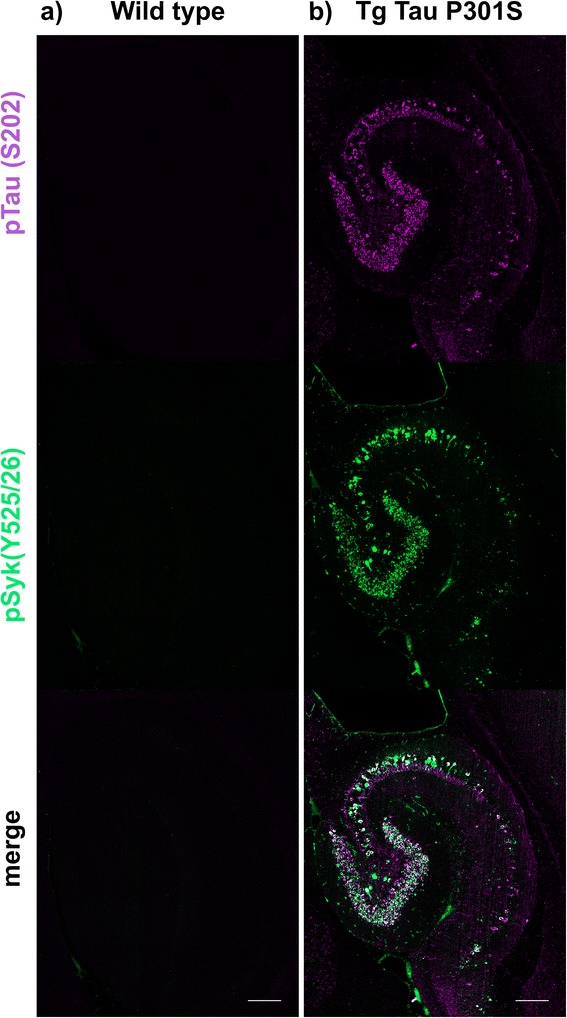
pSyk is increased in hippocampal neurons of Tg Tau P301S mice compared to wild-type littermates. Representative confocal image depicting 56 week-old male Tg Tau P301S and wild-type mice stained with antibodies against pTau (S202, purple) and pSyk (Y525/526, green). **a** Wild-type mice did not exhibit any tau phosphorylation nor Syk activation in their hippocampi. **b** Tau-overexpressing Tg Tau P301S mice exhibited a prominent tau phosphorylation at S202 that colocalized with Syk activation in hippocampal neurons. The scale bars represent 200 μm

Having shown that Aβ-overexpressing mouse models of AD exhibit an increased Syk activation in microglia and dystrophic neurites, we investigated whether Syk activation also occurs in Tg Tau P301S mice (a pure model of tauopathy) using immunofluorescence and confocal microscopy. Hippocampal neurons of Tg Tau P301S mice exhibit a high level of tau hyperphosphorylation (Fig. 4b) as well as an accumulation of pathogenic tau conformers (MC1, not shown) compared to WT littermates (Fig. 4a). Most importantly, pathological tau species clearly colocalize with pSyk (Y525/526) in hippocampal neurons (Fig. 4b). The pSyk burden is particularly prominent in hippocampal neurons of Tg Tau P301S mice (Fig. 4b) whereas WT littermates do not exhibit any pSyk immunoreactivity in the hippocampus (Fig. 4a).

**Fig. 5:**
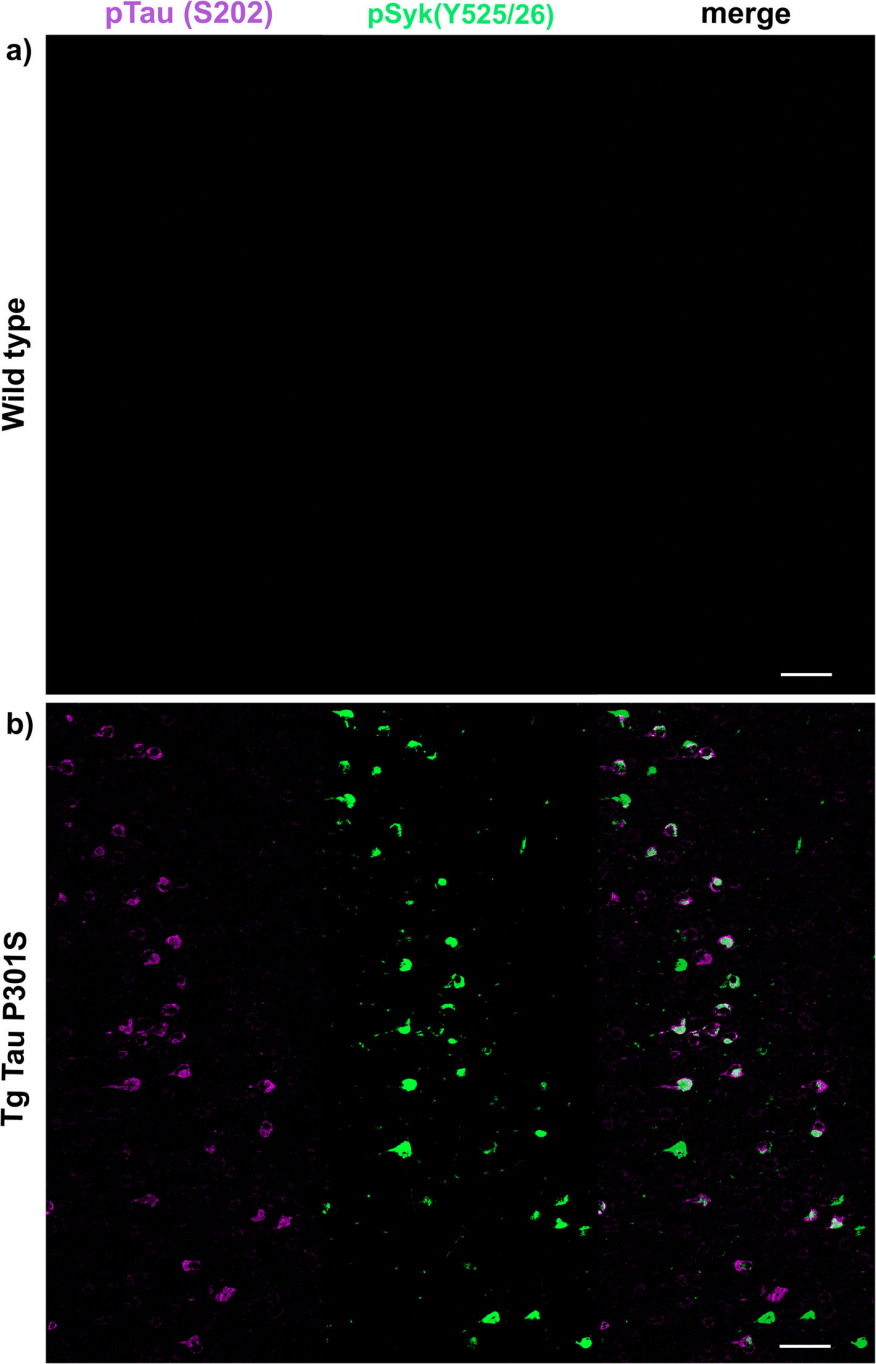
pSyk is increased in cortical neurons of Tg Tau P301S mice compared to wild-type littermates. Representative confocal image depicting 56 week-old male Tg Tau P301S and wild-type mice stained with antibodies against pTau (S202, purple), pSyk (Y525/526, *green*). **a** Wild-type mice did not exhibit any tau phosphorylation nor Syk activation in their cortices. **b** Tau-overexpressing Tg Tau P301S mice exhibited a prominent tau phosphorylation at S202 that colocalized with Syk activation in cortical neurons. The scale bars represent 100 μm

Cortical neurons of Tg Tau P301S mice also exhibit an increased level of tau hyperphosphorylation (Fig. 5b) compared to wild-type littermates (Fig. 5a). We observed a colocalization between pSyk and pTau (S202) immunoreactivities in cortical neurons. Interestingly, we also observed neurons that are singly immunopositive for tau or for pSyk. We addressed this observation by performing additional analyses (Figs. 8, 9, 10, 11, 12, 13, 14 and 15).

**Fig. 6:**
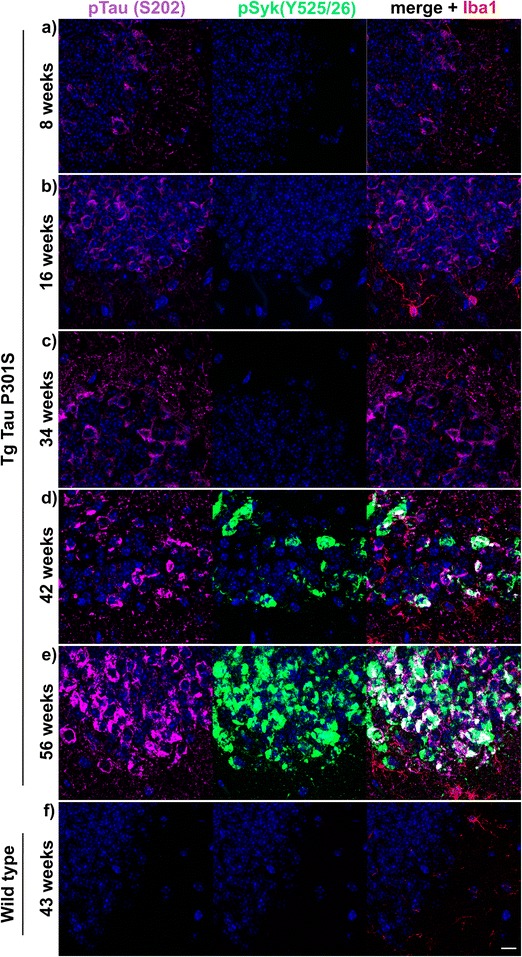
pSyk is increased age-dependently in hippocampal neurons of Tg Tau P301S mice compared to wild-type littermates. Representative confocal image depicting Tg Tau P301S and wild-type mice (*n* = 16) stained with antibodies against pTau (S202, *purple*), pSyk (Y525/526, *green*) and Iba-1 (*red*). Nuclei were stained with DAPI. **a**-**e** Tau-overexpressing Tg Tau P301S mice exhibited a prominent tau phosphorylation (S202) that increased with age and colocalized with Syk activation in hippocampal neurons. **f** Wild-type mice did not exhibit any tau phosphorylation nor Syk activation in their hippocampi. The scale bar represents 10 μm

**Fig. 7:**
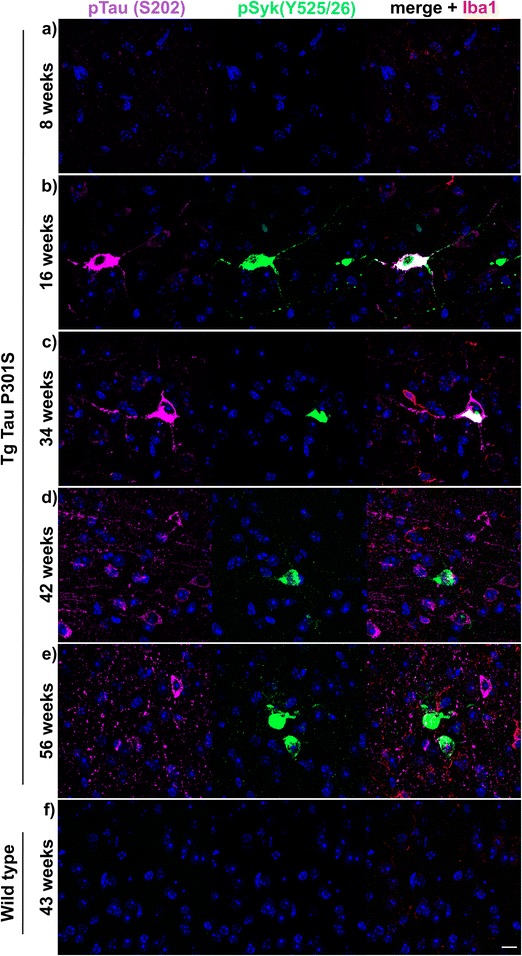
pSyk is increased age-dependently in cortical neurons of Tg Tau P301S mice compared to wild-type littermates. Representative confocal image depicting Tg Tau P301S and wild-type mice (*n* = 16) stained with antibodies against pTau (S202, *purple*), pSyk (Y525/526, *green*) and Iba-1 (*red*). Nuclei were stained with DAPI. **a**-**e** Tau-overexpressing Tg Tau P301S mice exhibited a prominent tau phosphorylation (S202) that increased with age and partially colocalized with Syk activation in cortical neurons. **f** Wild-type mice did not exhibit any tau phosphorylation nor Syk activation in their cortices. The scale bar represents 10 μm

We also analyzed the temporal changes of pSyk and tau levels in hippocampi and cortices of Tg Tau P301S mice between the age of 8 and 56 weeks (Figs. 6 and 7). WT mice do not exhibit any detectable tau phosphorylation (Fig. 6f) or tau oligomerization at any age (not shown). We then focused on the dentate gyrus of the hippocampus and found an age-dependent increase of tau phosphorylation (Fig. 6, S202, left panels) in Tg Tau P301S mice. Tau phosphorylation at S202 in the dentate gyrus was already detectable in 8-week-old Tg Tau P301S mice, however, pSyk immunoreactivity was not observed. Neurons immunopositive for pSyk (Y525/526) and pTau (S202) or tau conformers (MC1, not shown) were observed in the dentate gyrus of 42-week-old Tg Tau P301S mice (Fig. 6d). The neuronal pSyk burden also increases with age in Tg Tau P301S mice and is mainly restricted to the neuronal cell body (Fig. 6). Interestingly, microglia and neurites did not exhibit activated Syk in the hippocampus of Tau P301S mice (Fig. 6). Abnormal Syk activation seems to follow tau hyperphosphorylation (S202) in the hippocampus of Tg P301S mice (Fig. 6), as well as the formation of MC1-tau pathological conformers (data not shown here but MC1 and pSyk colocalization were quantified below).

Cortical neurons of Tg Tau P301S mice also show an increase in tau hyperphosphorylation and pSyk with age (Fig. 7). Interestingly, the onset of abnormal Syk activation occurs earlier (16 weeks of age) in the cortex than in the hippocampus (Fig. 7b compared to Fig. 6d). In conclusion, both pSyk and tau pathology in Tg Tau P301S mice increase with age but the progression is different in the hippocampus and the cortex. Many cortical neurons exhibit a colocalization of pSyk and pTau (S202) (Fig. 7b-c, e) but as mentioned earlier, there are also neurons that are singly immunopositive for pSyk or pTau.

**Fig. 8:**
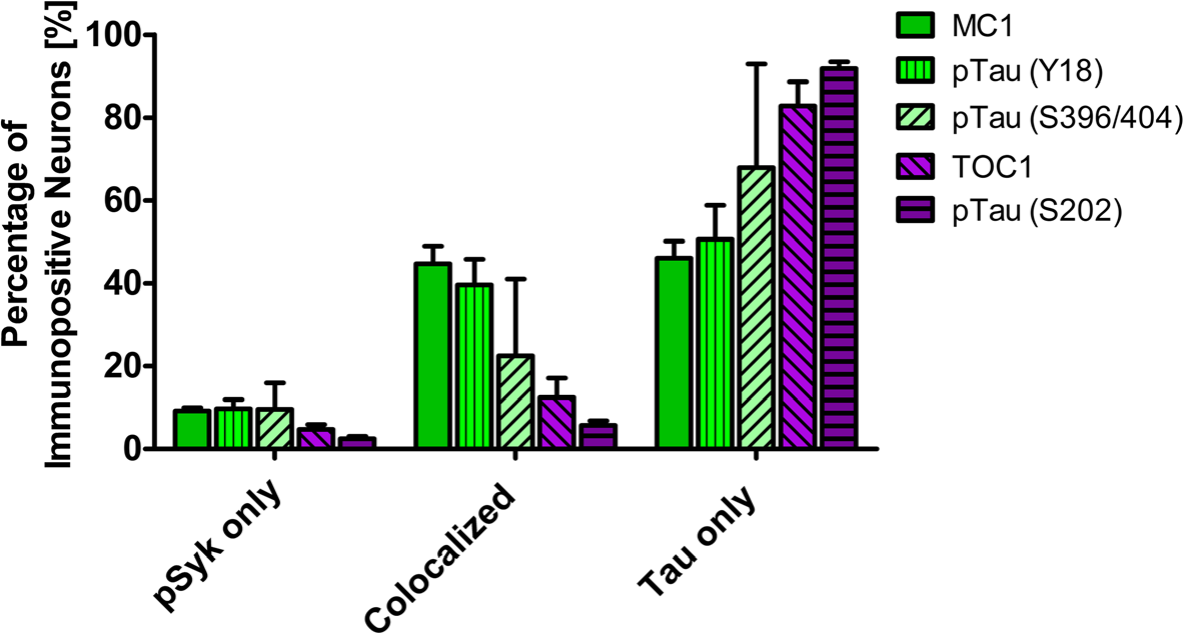
The degree of colocalization of pSyk and tau differs for various tau epitopes. Sections from Tg Tau P301S mice (*n* = 4, 47 ± 3.1-week-old) were stained with antibodies against pTau (S202, S396/404, Y18), tau oligomers (TOC1) or tau conformers (MC1) and pSyk (Y525/526, *green*). The cortices were divided in ROIs (each at a size of 50,000μm^2^) and neurons singly immunopositive for pSyk, singly immunoreactive for the respective tau epitope and the neurons immunopositive for both pSyk and the respective tau epitope (colocalized) were counted and the percentages of each neuronal fraction calculated using Zen *Blue* 2.1 (Zeiss) and Excel (MS Office), respectively. In average, 509 cortical fields were analyzed for each epitope (total of 21,800 neurons counted). The percentage of neurons singly immunopositive for pSyk was at a similar level for all tau epitopes investigated (pSyk only). MC1 and pTau Y18 show the highest colocalization with pSyk whereas the incidence of neurons immunopositive for both pSyk and TOC1 or pTau S202 was much lower (colocalized fraction). The error bars represent SEM

**Fig. 9:**
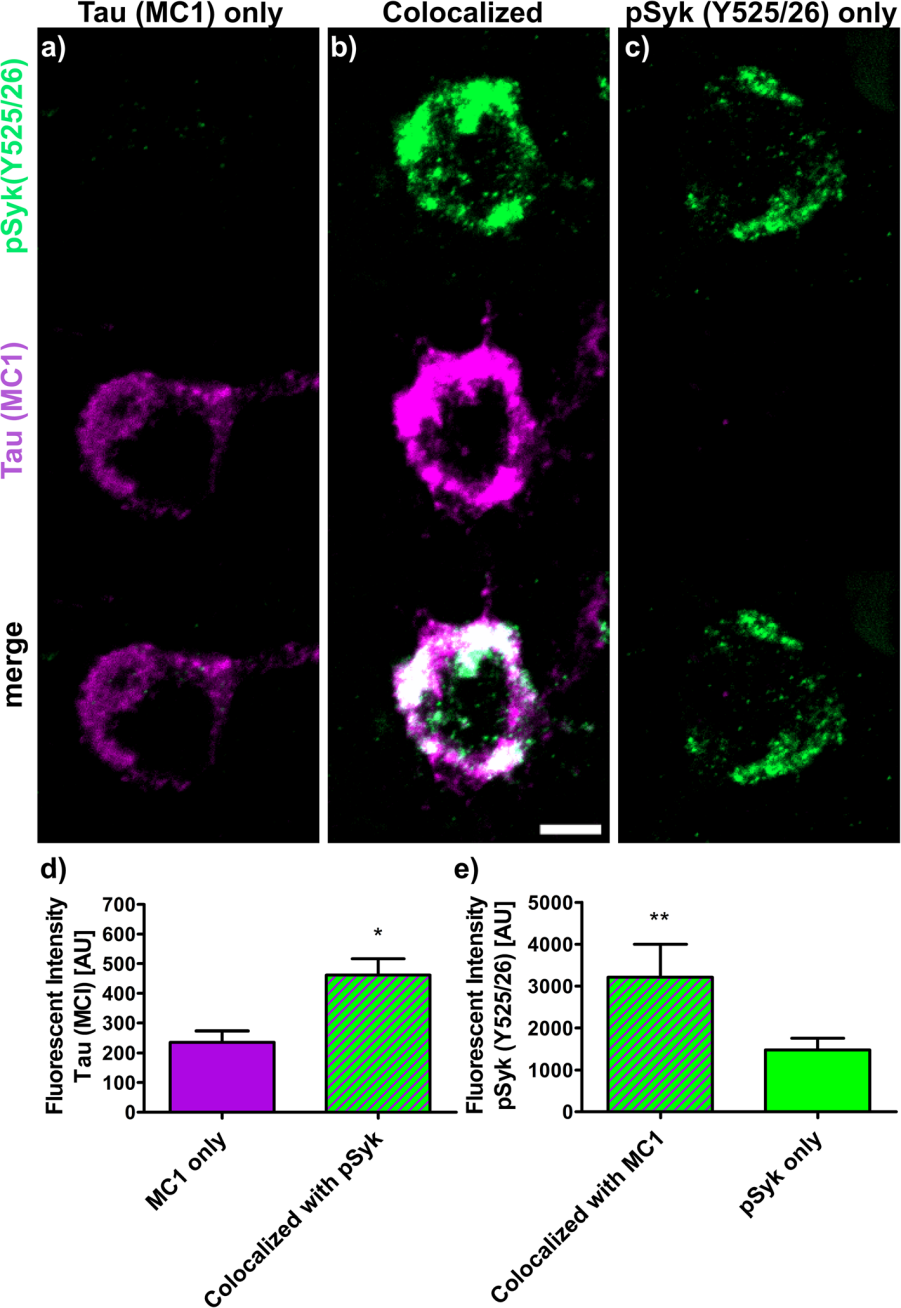
The amount of MC1 tau conformers and pSyk (Y525/526) levels cross-influence each other. Sections from Tg Tau P301S (*n* = 4, 47 ± 3.1-week-old) were stained with antibodies against tau conformers (MC1, *purple*) and pSyk (Y525/526, *green*). Fluorescent intensities of MC1 and pSyk were measured using Zen Blue 2.1 (Zeiss). Three different neuronal fractions were differentiated: **a** neurons singly immunopositive for MC1, (**b**) neurons singly immunopositive for pSyk and (**c**) neurons immunopositive for both MC1 and pSyk (colocalized). **d** Fluorescent intensities of MC1 were compared between neurons singly MC1 positive (*purple*) and neurons exhibiting a colocalization of MC1 and pSyk (*purple-green-striped*). Two-tailed Mann–Whitney test revealed a significant increase (*p* < 0.05) of MC1 fluorescent intensity in neurons exhibiting a colocalization of MC1 and pSyk compared to neurons singly immunopositive for MC1. **e** Fluorescent intensities of pSyk were compared between neurons singly immunopositive for pSyk (*green*) and neurons that show an immunoreactivity for both pSyk and MC1 (*purple-green-striped*). Two-tailed Mann–Whitney test revealed a significant increase (*p* < 0.01) of pSyk fluorescent intensity in neurons immunoreactive for both pSyk and MC1 compared neurons that are singly immunopositive for pSyk. The scale bar represents 10 μm. The error bars represent SEM

**Fig. 10:**
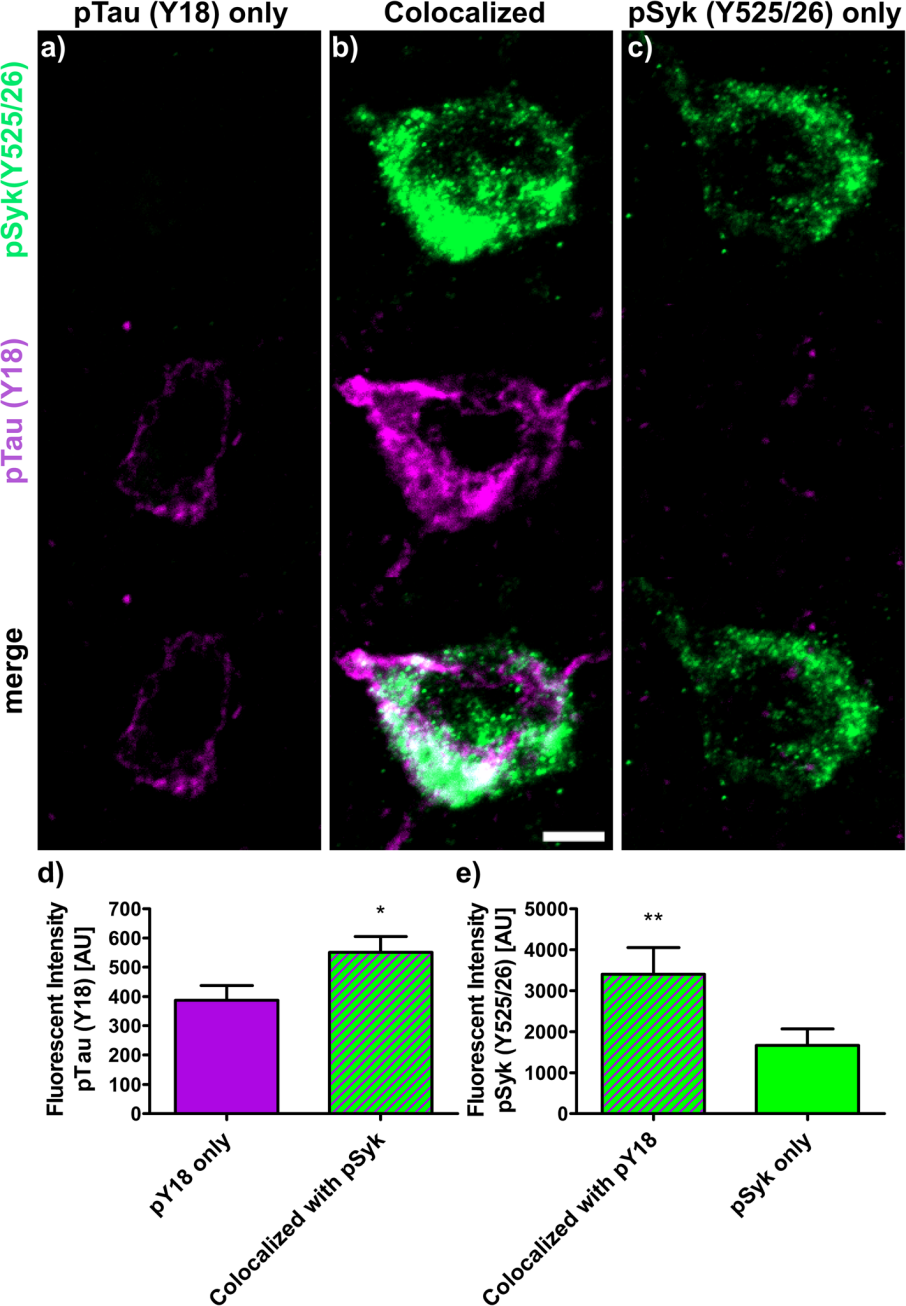
Tau phosphorylation at Y18 and Syk activation (Y525/526) cross-influence each other. Sections from Tg Tau P301S (*n* = 4, 47 ± 3.1-week-old) were stained with antibodies against phosphorylated tau (Y18, *purple*) and pSyk (Y525/526, *green*). Fluorescent intensities of pTau (pY18) and pSyk were measured using Zen Blue 2.1 (Zeiss). Three different neuronal fractions were differentiated: **a** neurons singly immunopositive for pY18, (**b**) neurons singly immunopositive for pSyk and (**c**) neurons immunopositive for both (colocalized). **d** Fluorescent intensities of pY18 tau were compared between singly pY18 immunopositive and the double immunopositive neurons (colocalized, purple-green-striped). Two-tailed Mann–Whitney test revealed a significant increase (*p* < 0.05) of pY18 fluorescent intensity for neurons exhibiting a colocalization compared to the neurons singly immunopositive for pY18 tau. **e** Fluorescent intensities of pSyk were compared between singly pSyk immunopositive neurons (*green*) and double immunopositive neurons for pSyk and pY18 tau (*purple-green-striped*). Two-tailed Mann–Whitney test revealed a significant increase (*p* < 0.01) of pSyk fluorescent intensity in neurons double immunopositive for pSyk and pY18 tau compared to singly pSyk immunopositive neurons. The scale bar represents 10 μm. The error bars represent SEM

**Fig. 11:**
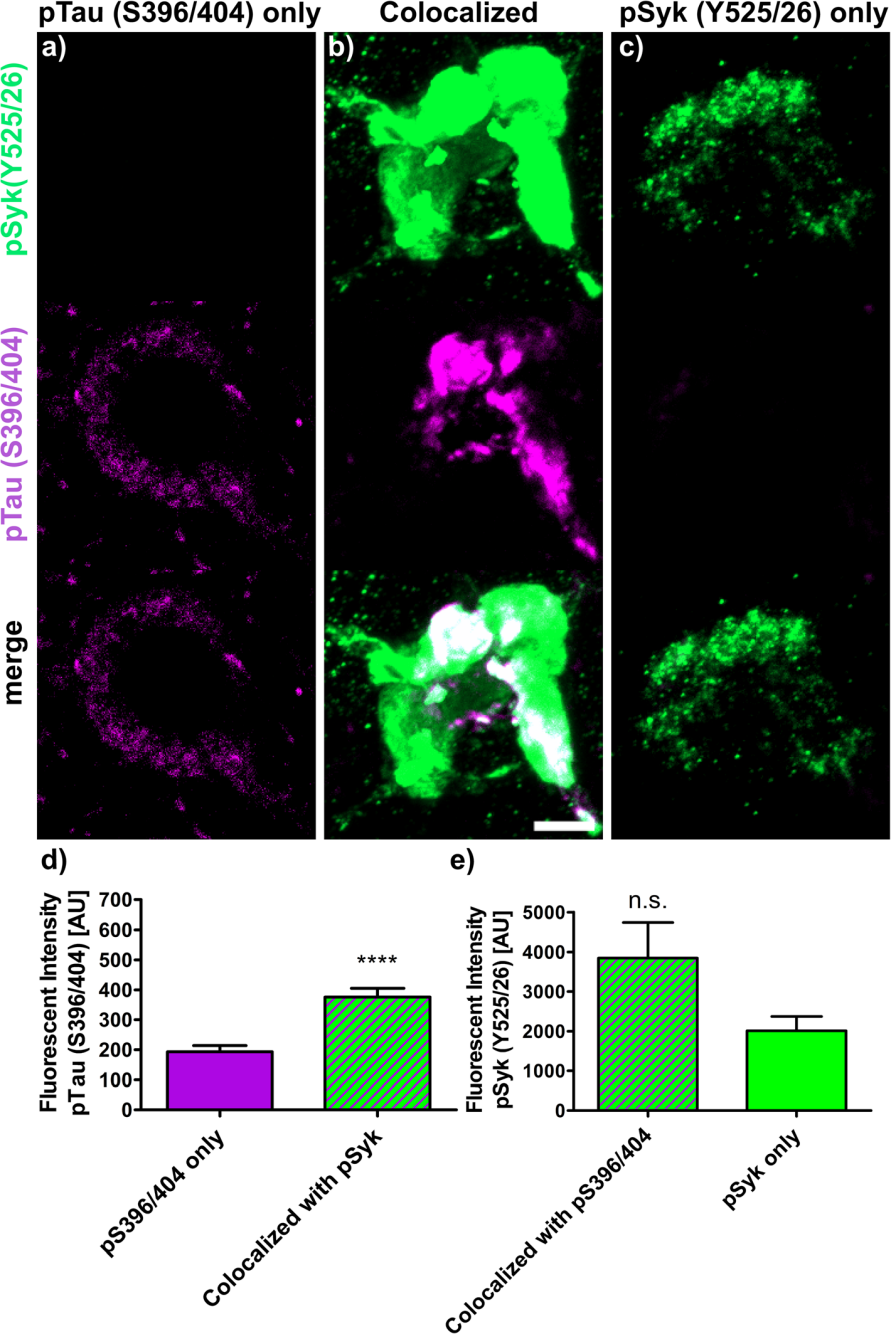
Syk activation (pSyk (Y525/526) influences the level of tau phosphorylation at S396/404. Sections from Tg Tau P301S (*n* = 4, 47 ± 3.1-week-old) were stained with antibodies against phosphorylated tau (S396/404, *purple*) and pSyk (Y525/526, *green*). Fluorescent intensities of pTau (pS396/404) and pSyk were measured using Zen Blue 2.1 (Zeiss). Three different neuronal fractions were differentiated: **a** neurons singly immunopositive for pS396/404 tau, (**b**) neurons singly immunopositive for pSyk and (**c**) neurons immunopositive for both (colocalized). **d** Fluorescent intensities of pS396/404 tau were compared between neurons singly immunopositive for pS396/404 (*purple*) and the neurons exhibiting a colocalization of pS396/404 and pSyk (*purple-green-striped*). Two-tailed Mann–Whitney test revealed a significant increase (*p* < 0.0001) of pS396/404 fluorescent intensity in the neurons exhibiting a colocalization compared to neurons singly immunopositive for pS396/404 tau. **e** Fluorescent intensities of pSyk were compared between singly pSyk immunopositive neurons (*green*) and neurons exhibiting a colocalization of pSyk and pS396/404 tau (*purple-green-striped*). Two-tailed Mann–Whitney test revealed no significant change of pSyk fluorescent intensity in neurons exhibiting a colocalization compared to singly pSyk immunopositive neurons. The scale bar represents 10 μm. The error bars represent SEM

**Fig. 12:**
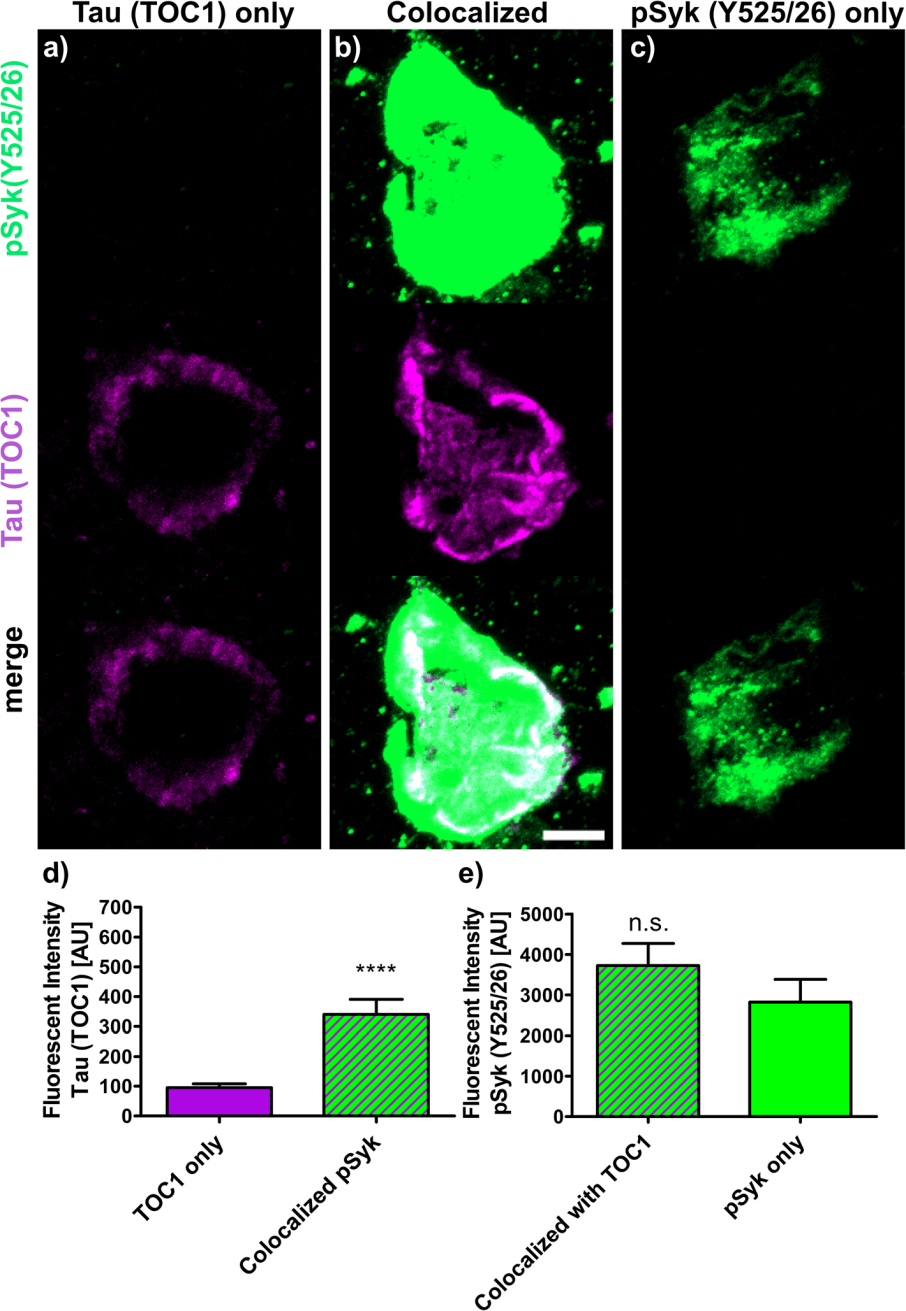
Syk activation (Y525/526) influences the level of tau oligomerization (TOC1). Sections from Tg Tau P301S (*n* = 4, 47 ± 3.1-week-old) stained with antibodies against tau oligomers (TOC1, *purple*) and pSyk (Y525/526, *green*). Fluorescent intensities of oligomerized tau (TOC1) and pSyk were measured using Zen Blue 2.1 (Zeiss). Three different neuronal fractions were differentiated: **a**) neurons singly immunopositive for TOC1, (**b**) neurons singly immunopositive for pSyk and (**c**) neurons immunopositive for both (colocalized). **d** Fluorescent intensities of TOC1 were compared between neurons singly immunopositive for TOC1 (*purple*) and neurons exhibiting a colocalization of pSyk and TOC1 (*purple-green-striped*). Two-tailed Mann–Whitney test revealed a significant increase (*p* < 0.0001) of TOC1 fluorescent intensity in neurons exhibiting a colocalization with pSyk compared to singly TOC1 immunopositive neurons. **e** Fluorescent intensities of pSyk were compared between neurons singly immunopositive for pSyk (*green*) and neurons exhibiting a colocalization pf TOC1 and pSyk (*purple-green-striped*). Two-tailed Mann–Whitney test revealed no significant change of pSyk fluorescent intensity in neurons exhibiting a colocalization compared to the singly pSyk immunopositive neurons. The scale bar represents 10 μm. The error bars represent SEM

**Fig. 13:**
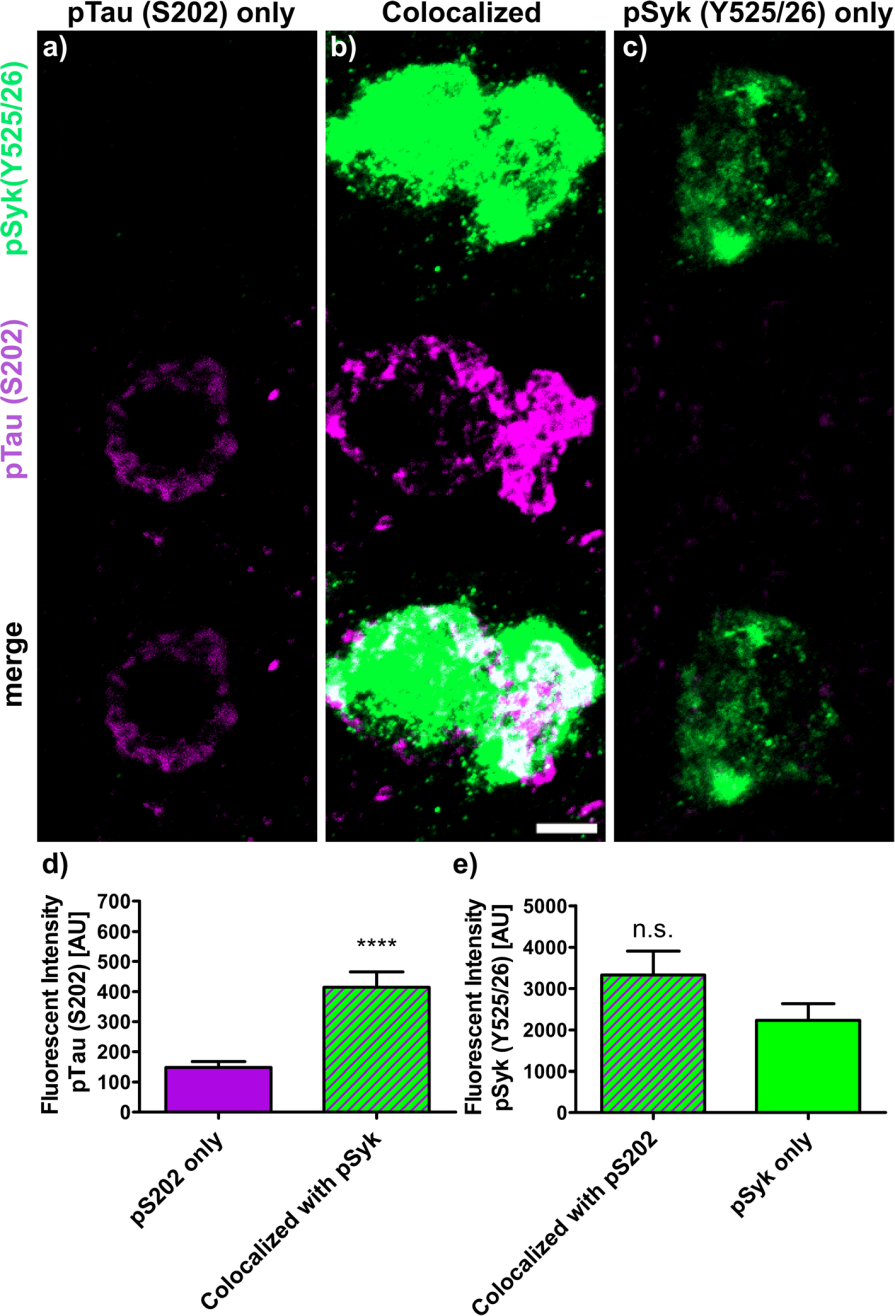
Syk activation (Y525/526) influences the level of tau phosphorylation at S202. Sections from Tg Tau P301S (*n* = 4, 47 ± 3.1-week-old) stained with antibodies against phosphorylated tau (S202, *purple*) and pSyk (Y525/526, *green*). Fluorescent intensities of pTau (S202) and pSyk were measured using Zen Blue 2.1 (Zeiss). Three different neuronal fractions were differentiated: **a** neurons singly immunopositive for S202, (**b**) neurons singly immunopositive for pSyk and (**c**) neurons immunopositive for both (colocalized). **d** Fluorescent intensities of pS202 tau were compared between neurons singly pS202 immunopositive (*purple*) and neurons exhibiting a colocalization between pS202 and pSyk (*purple-green-striped*). Two-tailed Mann–Whitney test revealed a significant increase (*p* < 0.0001) of pS202 fluorescent intensity in neurons exhibiting a colocalization compared to neurons singly immunopositive for pS202. **e** Fluorescent intensities of pSyk were compared between neurons singly immunopositive for pSyk (*green*) and neurons exhibiting a colocalization of pSyk and pS202 tau (*purple-green-striped*). Two-tailed Mann–Whitney test revealed no significant change of pSyk fluorescent intensity in neurons exhibiting a colocalization compared to singly pSyk immunopositive neurons. The scale bar represents 10 μm. The error bars represent SEM

We further quantified the number of neurons that are singly pSyk immunopositive, singly immunopositive for tau pathogenic species and neurons immunopositive for both pSyk and tau pathogenic species in the cortex of 47-week-old Tg Tau P301S mice (Fig. 8). We calculated the percentages of neurons singly immunopositive for either pSyk, pathogenic tau species or neurons immunopositive for both. The sum of all cortical neurons counted was considered 100% including neurons positive for pSyk and the respective tau epitope and neurons immunopositive for both. For all the tau epitopes tested, we found that only a small fraction of the neurons is singly immunopositive for pSyk (9.7 ± 4.4% (pTau, Y18); 2.5 ± 1.2% (pTau, S202); 9.2 ± 1.6% (MC1 pathogenic tau conformers); 9.6 ± 6.3% (pTau, S396/404); 4.8 ± 2.0% (TOC1 (tau oligomers)). Interestingly, a larger percentage of neurons is immunoreactive for both pSyk and tau pathogenic species (44.7 ± 8.6% (MC1); 39.7 ± 12.4% pTau Y18; 22.5 ± 18.6% (PHF-1, pTau S396/404); 12.4 ± 8.1% (TOC1, tau oligomers) but only 5.7 ± 2.2% for pTau (S202)). The neurons singly immunopositive for tau complement these observations with relative values of 46.1 ± 8.2% (MC1), 50.6 ± 16.3% (Y18), 67.9 ± 24.9% (S396/404), 82.8 ± 10.1% (TOC1), and 91.8 ± 3.2% (S202) (Fig. 8). The differences in relative colocalization between pSyk and specific tau pathologic species suggest that specific pathogenic forms of tau may have a different impact on Syk activation or either that Syk activation may contribute to the formation of specific tau pathogenic species (Fig. 8). We therefore subsequently measured the fluorescent intensities of pSyk and of the different tau epitopes to determine whether the level of Syk activation correlates with the amount of specific tau pathogenic species. In general, we found that neurons that exhibit a high level of pSyk immunoreactivity also demonstrate a higher level of tau pathogenic species whereas neurons that are weakly immunopositive for pSyk show less tau pathology (Figs. 9, 10, 11, 12 and 13). In addition, neurons that are singly immunopositive for tau pathogenic species (including hyperphosphorylated tau and misfolded tau) show also less intense tau pathologies, as measured by fluorescent intensities, than neurons that are displaying both pSyk and tau pathology, further supporting a role of Syk in the formation of tau pathogenic species. For instance, the level of pathogenic tau conformers (MC1) is significantly increased in neurons that are also strongly immunopositive for pSyk compared to neurons that are singly immunopositive for MC1 (Fig. 9d, *p* < 0.05). Interestingly, the level of pSyk is also significantly increased in neurons that display an accumulation of MC1 pathogenic conformers compared to neurons that are singly immunopositive for pSyk (Fig. 9e, *p* < 0.01). These data suggest that pathogenic tau conformers and Syk activation may promote each other. We found that tau phosphorylation at Y18 is significantly increased in neurons that are also immunopositive for pSyk (Fig. 10d, *p* < 0.05) which is consistent with previous data showing that in vitro Syk can phosphorylates tau at Y18. We have previously shown that Syk positively regulates GSK-3β activity in vitro. It is therefore consistent with our observation that the GSK-3β-dependent phospho-tau epitope (S396/404, PHF-1) is also increased in neurons that display Syk activation (Fig. 11d, *p* < 0.0001). The pSyk level, however, is not statistically significantly increased in neurons that are immunopositive for both PHF-1 and pSyk compared to neurons that are singly immunopositive for pSyk suggesting that PHF-1 phosphorylated tau species do not induce Syk activation (Fig. 11e). Similar observations were obtained for tau oligomers (Fig. 12, TOC1) and tau species phosphorylated at S202 (Fig. 13, CP13). Altogether, these data suggest that only certain pathogenic forms of tau (MC1, Y18) promote Syk activation, whereas Syk activation appears to directly induce tau phosphorylation at Y18 and to indirectly regulate tau phosphorylation at multiple epitopes (S396/404, S202) as well as tau misfolding (MC1, TOC1).

### Syk overexpression increases tau phosphorylation and total tau levels in SH-SY5Y cells

**Fig. 14:**
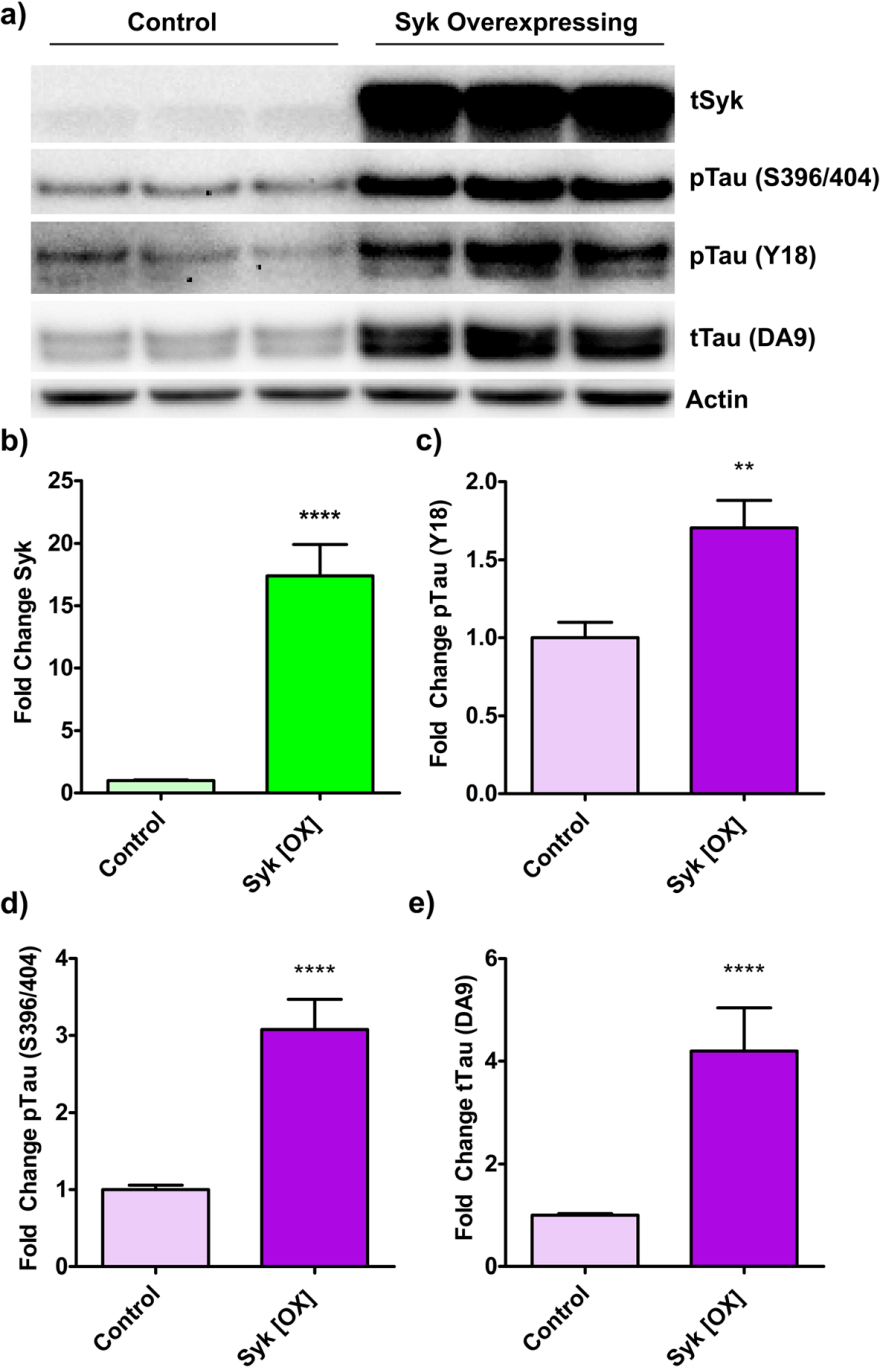
Syk overexpression increases tau phosphorylation and total tau levels in SH-SY5Y cells. SH-SY5Y cells were transfected with either the empty plasmid as a control or with the same plasmid carrying the Syk sequence for overexpression (Syk OX). Proteins expressed by transfected SH-SY5Y cells were analyzed by Western-blotting. Western-blots chemiluminescent signals were quantified and results were tested for normal distribution using the Shapiro-Wilk test. A subsequent Mann–Whitney test was used to test for statistical significance. **a** Representative Western blots are shown. **b** Level of Syk overexpression is in average 17.3 times higher than in control cells (*p* ≤ 0.0001, *n* = 18). **c** Level of tau phosphorylation at Y18 is 1.7 times higher in Syk overexpressing compared to control cells (*p* ≤ 0.01, *n* = 11). **d** Level of tau phosphorylation at S396/404 is 3 times higher in Syk overexpressing compared to control cells (*p* ≤ 0.0001, *n* = 17). **e** Level of total tau (DA9) is 4.1 times higher in Syk overexpressing compared to control cells (*p* ≤ 0.0001, *n* = 18). The error bars represent SEM

To further investigate the impact of Syk on tau, we generated human neuronal-like (SH-SY5Y) cells overexpressing Syk (Syk-OX). Syk-OX SH-SY5Y cells show an approximate 17-fold increase in Syk expression compared to control SH-SY5Y cells transfected with the empty vector (Fig. 14b, p < 0.0001). Interestingly, Syk upregulation in SH-SY5Y cells leads to a significant increase (1.7-fold) in phosphorylated tau at Y18 (Fig. 14c, p < 0.01) and at S396/404 (Fig. 14d, 3-fold, *p* < 0.0001) compared to control cells. Total tau levels are also significantly increased following Syk overexpression (Fig. 14e, 4.2-fold, *p* < 0.0001). We analyzed the possible impact of Syk overexpression on Tau mRNA levels by quantitative RT-PCR and found that Syk overexpression does not affect Tau transcription (data not shown) suggesting that Syk may regulate tau degradation or tau protein translation. In summary, these results show that the accumulation of tau pathogenic species can trigger Syk activation, as shown in Tg Tau P301S mice (Figs. 8, 9, 10, 11, 12 and 13), whereas Syk itself appears to regulate total tau levels and tau phosphorylation at multiple epitopes (Fig. 14) therefore influencing the development of the tau pathology.

### Syk activity is increased in cortical neurons immunopositive for pTau (Y18), conformationally altered Tau (MC1) and in dystrophic neurites in human AD compared to non-demented control

**Fig. 15:**
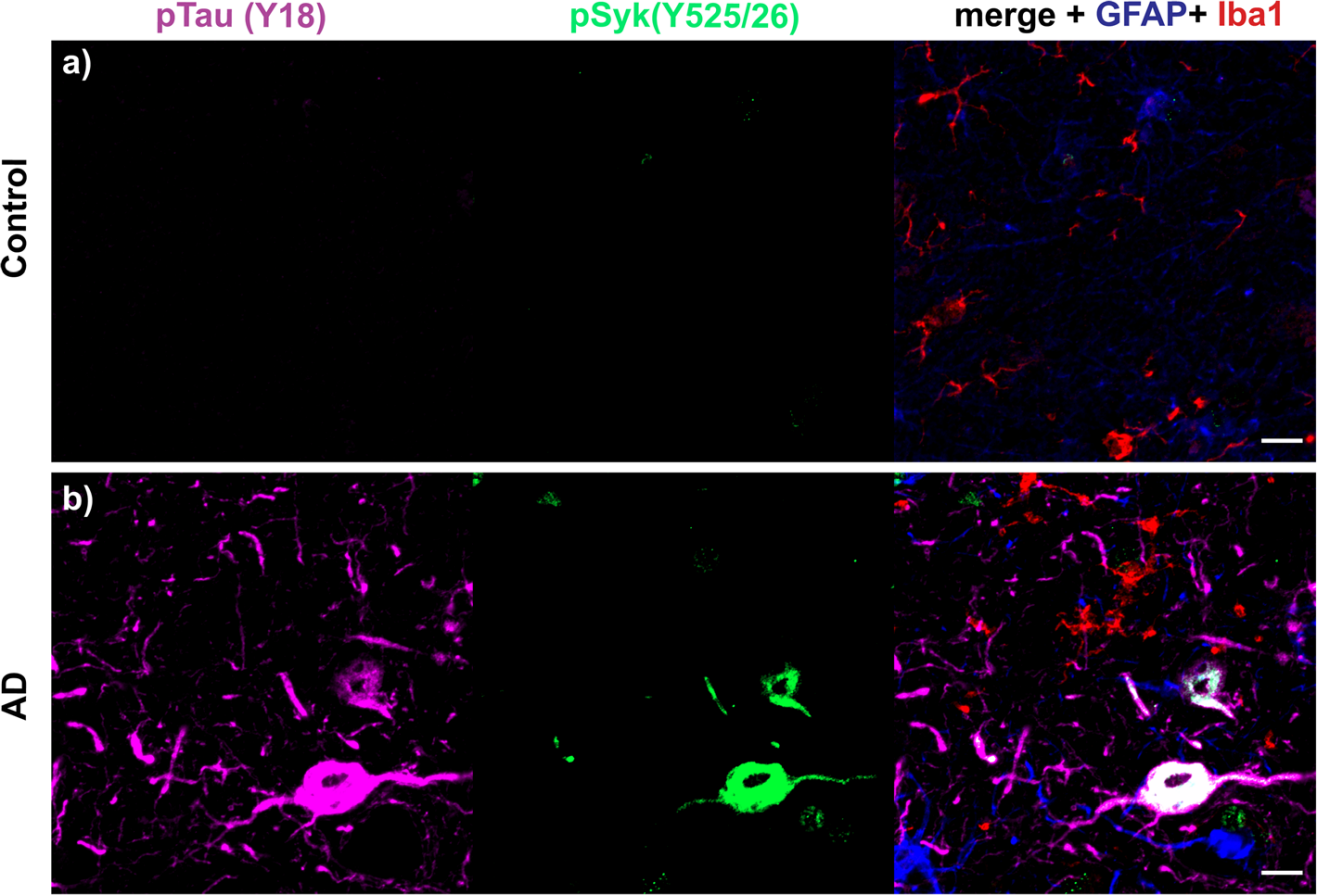
pSyk is increased in cortical neurons immunopositive for pTau (Y18) of human AD compared to non-demented controls. Representative confocal images of the dorsolateral frontal cortex sections of human AD (**b**) were stained with antibodies against pTau (Y18) (*purple*), pSyk (525/526) (*green*), Iba-1 (*red*) and GFAP (*blue*) and compared to control brain sections (**a**). **a** The non-demented control (102-year-old, male) does not exhibit any tau phosphorylation nor increased Syk activation in the dorsolateral frontal cortex. **b** The AD brain (67-year-old, male) exhibits a prominent tau phosphorylation at Y18 that colocalizes with Syk activation (Y525/526) in cortical neurons. The scale bars represent 10 μm

**Fig. 16:**
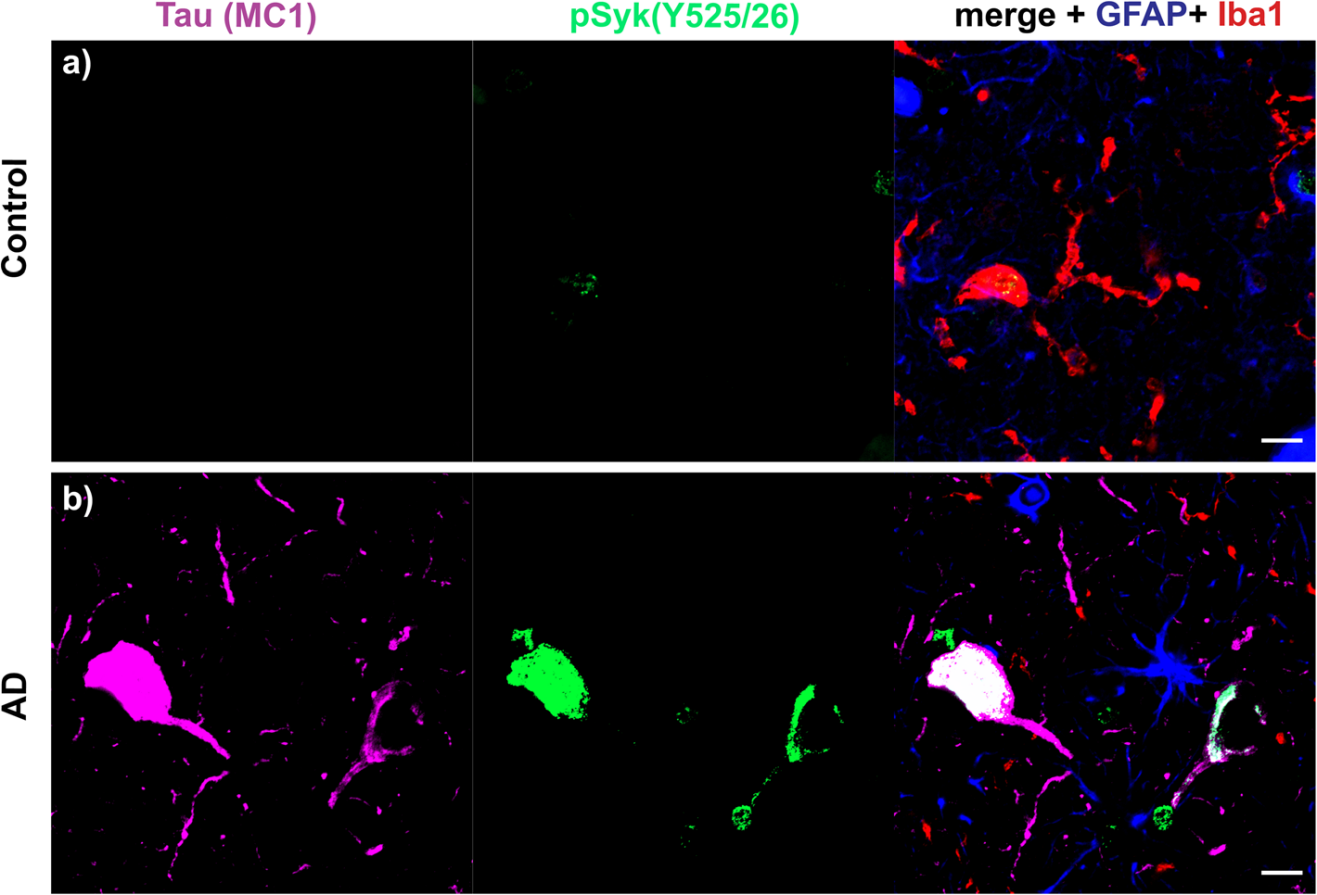
pSyk is increased in cortical neurons immunopositive for MC1 pathogenic Tau conformers in AD compared to brain sections from a non-demented control. Representative confocal images of the dorsolateral frontal cortex sections of human AD (**b**) were stained with antibodies against conformationally altered tau species (MC1) (*purple*), pSyk (525/526) (*green*), Iba-1 (*red*) and GFAP (*blue*) and compared to non-demented control brain sections (**a**). **a** The non-demented control (102-year-old, male) does not exhibit any cells immunopositive for MC1 nor increased in Syk activation in the dorsolateral frontal cortex. **b** The AD brain (67-year-old, male) exhibits neurons strongly immunopositive for MC1 that colocalize with Syk activation (Y525/526) in cortical neurons. The scale bars represent 10 μm

**Fig. 17:**
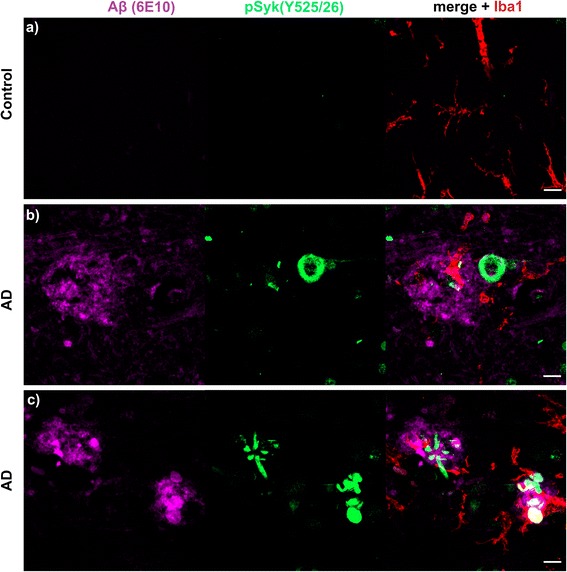
pSyk is increased in dystrophic neurites associated with β-amyloid plaques of human AD patients compared to healthy controls. Representative confocal images of dorsolateral frontal cortex sections of human AD (**b**) were stained with antibodies against Aβ (6E10) (*purple*), pSyk (525/526) (*green*), Iba-1 (*red*) and compared to non-demented control brain sections (**a**). **a** The non-demented control (102-year-old, male) does not exhibit any Aβ deposits nor increased Syk activation in neurons. **b** The AD brain (67-year-old, male) exhibits neurons strongly immunopositive for pSyk (Y525/526). **c** The AD brain also exhibits dystrophic neurites immunopositive for pSyk (Y525/526) near/within Aβ deposits. The scale bars represent 10 μm

We also performed different immunostainings against Aβ, pSyk, GFAP, Iba-1, tau pathogenic conformers (MC1) and phosphorylated tau at Y18 using brain sections from human AD and non-demented controls. We found an increase in Syk activation in DNs surrounding Aβ deposits as well as in neurons displaying an accumulation of phosphorylated Tau at Y18 and elevated levels of MC1 pathogenic tau conformers in AD brain sections whereas only weak immunoreactivity for pSyk was observed in brain sections from a non-demented control (Figs. 15, 16 and 17). As observed in the AD mouse models, astrocytes did not exhibit Syk activation in neither the AD brain section nor the control. Only a subset of microglial cells exhibited a weak pSyk signal. Most of the detected pSyk signal was of neuronal origin and either localized in somata or DNs. These data complement our observation in AD mouse models and reveal an association between Syk activation and typical AD pathological lesions in the human brain. Further studies will be required using a larger sample of AD pathological specimen to further clarify the role of Syk activation in AD brains.

## Discussion

Our previous studies have shown that tau hyperphosphorylation, Aβ production and neuroinflammation are reduced following Syk inhibition [28]. These data prompted us to investigate the level of Syk activation in different mouse models of AD and in brain sections from a non-demented control and an AD patient. We found that Syk activation occurs in three different mouse models of AD, overexpressing Aβ or tau, showing that Syk activation is triggered by both Aβ deposits and tau pathological species. Most importantly, we made similar observations in human AD brain sections.

Recent late phase clinical trials targeting the major pathological hallmarks of AD, mainly extracellular Aβ plaques or intra-neuronal tau aggregates, have been unsuccessful so far and have failed to prevent cognitive decline and brain atrophy in AD patients [7, 19, 37, 39]. As PET scan imaging of AD patients reveals that Aβ deposits and pathological tau accumulation occur during the prodromal phase of AD [26], it has been suggested that therapies that are targeting Aβ or pathological tau accumulation must be implemented decades before the appearance of the symptoms to be successful [26]. Hence, pharmacological intervention at downstream targets of Aβ and tau may represent a more promising therapeutic strategy for AD patients. However, therapeutic targets downstream of the Aβ and tau pathological lesions remain to be identified. Our work supports the view that Syk may be such a therapeutic target as it appears to be activated in vivo in response to β-amyloidosis and the formation of pathological tau species.

In this study, we report a hyperactivation of Syk in the brains of three different AD mouse models versus wild-type/littermate controls and human AD compared to non-demented controls. In Tg PS1/APPsw, Tg APPsw mice, Syk activity is largely increased in activated microglia and in DNs around Aβ deposits. In addition, we observed an activation of Syk in DNs around Aβ deposits in an AD pathological specimen. In Tg Tau P301S mice and AD brain sections, Syk hyperactivation is colocalized with misfolded tau and hyperphosphorylated tau in neurons.

The strong increase in activated Syk observed in dystrophic neurites (DNs) surrounding Aβ deposits may suggest the involvement of Syk in the formation of these DNs that ultimately leads to the synaptic loss observed in AD [32]. DNs are characterized by an accumulation of BACE-1 and sAPPβ which implies a contribution of DNs to Aβ production and accumulation [31]. In fact, several in vivo studies have shown that BACE-1 immunopositive dystrophic neurites precede Aβ plaque formation in the brains of 3xTg-AD, 2xFAD and 5xFAD mice and therefore, represent an early pathological event in AD [2, 16, 45]. Our previous in vitro and in vivo data have shown that Syk regulates Aβ and sAPPβ production via a modulation of BACE-1 expression [28] and therefore support a causative role of Syk activation in the accumulation of BACE-1 and sAPPβ in DNs.

The increased activation of Syk in activated microglia of Aβ-overexpressing mice further supports a role of Syk in microglial activation in vivo and suggests that Aβ accumulation can lead to an activation of Syk in microglia. Previous in vitro studies have shown that Aβ fibrils and oligomers can trigger a microglial inflammatory response mediated by Syk and leading to neurotoxicity [3, 4, 23].

Recruitment and activation of Syk can also be mediated by activation of triggering receptor expressed on myeloid cells 2 (TREM2) [18]. TREM2 is a type I transmembrane protein and part of the immunoglobulin (Ig) receptor superfamily. Since TREM2 does not have any cytoplasmic signaling motifs, an adaptor protein DNAX-activating protein of 12 kDa (DAP12, also known as TYROBP) is needed for TREM2 signal transduction. DAP12 interacts with the transmembrane domain of TREM2. The cytoplasmic domain of DAP12 contains an immunoreceptor tyrosine activation motif (ITAM) that provides docking sites for Syk activation. Interestingly, loss-of-function mutations in the DAP12 or TREM2 genes cause a rare autosomal recessive disorder called Nasu-Hakola disease (NHD) whereas heterozygous carriers of these mutations show an elevated risk to develop AD [27]. Symptoms of NDH include multifocal bone cysts and presenile dementia. Interestingly, Syk activation (pSyk, Y525/526) is increased in NHD neurons compared to controls [33] and was found to be also present in microglia and macrophages but not in astrocytes or oligodendrocytes [33] supporting a role of Syk activation in the development of NHD dementia.

Syk plays a key role in the activation of immune cells and the production of inflammatory cytokines. We have shown previously that activation of NFκB (nuclear factor kappa-light-chain-enhancer of activated B cells) which is known to play a regulatory role in neuroinflammation, is prevented following either pharmacological Syk inhibition or genetic knockdown of Syk [28]. Hence, this suggests a role of Syk in the regulation of neuroinflammation. In addition, Syk has been shown to mediate the neuroinflammation and neurotoxicity caused by Aβ [3, 23]. Furthermore, the Aβ-induced cytokine production by microglia has been found to be mediated by Syk [4], suggesting that Syk is involved in the microglial proinflammatory response.

The pathological analysis of Tg Tau P301S mice shows that Syk activation is associated with the formation of hyperphosphorylated tau and misfolded tau in the hippocampus and cortex while our previous work has shown that Syk inhibition can reduce tau phosphorylation at multiple AD relevant epitopes [28]. Interestingly, we show here that Syk upregulation in human neuronal like SH-SY5Y cells induces tau accumulation and tau phosphorylation further confirming a role of Syk in the formation of tau pathogenic species. Altogether, our data suggest that Syk activation may also promote tau hyperphosphorylation and misfolding in vivo as neurons that show higher levels of Syk activation also show more accumulation of hyperphosphorylated tau and tau pathogenic conformers. Pathological tau species accumulation clearly results in Syk activation in Tg Tau P301S mice while Syk activation appears to be a mediator of the formation of tau pathogenic species, thereby implying the existence of a positive feedback loop resulting in an enhanced progression of tau pathology. Given that Syk is also present in DNs which exhibit tau accumulation and tau phosphorylation [35, 40], this further supports a pathological role of Syk in the formation of DNs and ultimately synaptical loss.

Our previous in vivo and in vitro data show decreased tau phosphorylation at multiple epitopes (S396/404, S202, Y18) following Syk inhibition [28]. Interestingly, we show here that Syk overexpression in SH-SY5Y cells increases tau phosphorylation and total tau levels (Y18, S396/404, DA9). The increase in total tau levels following Syk upregulation is not caused by an increased transcription, as tau mRNA levels do not vary between Syk overexpressing and control cells (data not shown). Therefore, increased Syk levels may lead to an increased translation or decreased degradation of tau or a combination of both. However, the molecular mechanisms responsible for the increased tau levels following Syk overexpression or decreased tau following Syk inhibition remain to be further investigated and are currently being studied in our laboratory.

In this study, we also provide evidence for an aberrant Syk activation in dystrophic neurites around Aβ deposits and in neurons immunopositive for pathological tau species in human AD brain sections further validating the data obtained with different transgenic mouse models of AD.

## Conclusions

In conclusion, our data support a pathological role of Syk in the formation of Aβ deposits and misfolded tau and suggest additionally that reduction of Syk hyperactivity through pharmacological inhibition may be a promising therapeutic approach for the treatment of AD.
